# Green fabrication of chitosan nanoparticles using *Lavendula angustifolia*, optimization, characterization and in‑vitro antibiofilm activity

**DOI:** 10.1038/s41598-023-37660-6

**Published:** 2023-07-10

**Authors:** Noura El-Ahmady El-Naggar, Marwa Eltarahony, Elsayed E. Hafez, Shimaa I. Bashir

**Affiliations:** 1 Department of Bioprocess Development, Genetic Engineering and Biotechnology Research Institute, City of Scientific Research and Technological Applications (SRTA-City), New Borg El-Arab City, Alexandria, 21934 Egypt; 2 Environmental Biotechnology Department, Genetic Engineering and Biotechnology Research Institute (GEBRI), City of Scientific Research and Technological Applications (SRTA-City), New Borg El-Arab City, Alexandria, 21934 Egypt; 3 Department of Plant Protection and Biomolecular Diagnosis, Arid Land Cultivation Research Institute, City of Scientific Research and Technological Applications (SRTA-City), New Borg El‑Arab City, Alexandria, 21934 Egypt

**Keywords:** Nanobiotechnology, Nanoparticles

## Abstract

Chitosan nanoparticles (CNPs) are promising polymeric nanoparticles with exceptional physicochemical, antimicrobial and biological characteristics. The CNPs are preferred for a wide range of applications in the food industry, cosmetics, agriculture, medical, and pharmaceutical fields due to their biocompatibility, biodegradability, eco-friendliness, and non-toxicity. In the current study, a biologically based approach was used to biofabricate CNPs using an aqueous extract of *Lavendula angustifolia* leaves as a reducing agent. The TEM images show that the CNPs were spherical in shape and ranged in size from 7.24 to 9.77 nm. FTIR analysis revealed the presence of several functional groups, including C–H, C−O, CONH_2_, NH_2_, C–OH and C–O–C. The crystalline nature of CNPs is demonstrated by X-ray diffraction. The thermogravimetric analysis revealed that CNPs are thermally stable. The CNPs' surface is positively charged and has a Zeta potential of 10 mV. For optimising CNPs biofabrication, a face-centered central composite design (FCCCD) with 50 experiments was used. The artificial intelligence-based approach was used to analyse, validate, and predict CNPs biofabrication. The optimal conditions for maximum CNPs biofabrication were theoretically determined using the desirability function and experimentally verified. The optimal conditions that maximize CNPs biofabrication (10.11 mg/mL) were determined to be chitosan concentration 0.5%, leaves extract 75%, and initial pH 4.24. The antibiofilm activity of CNPs was evaluated in‑vitro. The results show that 1500 μg/mL of CNPs suppressed *P. aeruginosa*, *S. aureus* and *C. albicans* biofilm formation by 91.83 ± 1.71%, 55.47 ± 2.12% and 66.4 ± 1.76%; respectively. The promising results of the current study in biofilm inhibition by necrotizing biofilm architecture, reducing its significant constituents and inhibiting microbial cell proliferation encourage their use as natural biosafe and biocompatible anti-adherent coating in antibiofouling membranes, medical bandage/tissues and food packaging materials.

## Introduction

Chitosan is a naturally occurring polymeric, cationic polysaccharide derived from chitin via partial deacetylation. It comprises N-acetyl glucosamine and (1 → 4)-linked glucosamine residues that are randomly dispersed throughout its structure^1^. Chitosan nanoparticles (CNPs) are promising bio-based nanoparticles that are easy to prepare, less toxic, biodegradable, biocompatible, and have tremendous potential for various applications. Bulk materials' properties remain relatively constant regardless of their size. However, nanoparticles could have superior capabilities due to their unique characteristics, which include their small size and quantum size effects^2^.

Nowadays, CNPs are promising candidates for various applications, including medical applications, biomedical engineering, agriculture, and the pharmaceutical industries^3–7^. Chitosan has been extensively applied as a topical dressing in wound healing due to its antimicrobial, healing-promoting, nontoxic, hemostatic, biodegradable, and biocompatible characteristics^8^. CNPs used as an efficient system to deliver vaccines, such as influenza antigens, to stimulate protective immunity^9^. CNPs show effective antimicrobial activity against *Escherichia coli*, *Staphylococcus aureus*, *Pseudomonas aeruginosa*, *Klebsiella pneumoniae* and pathogenic multidrug-resistant bacteria, *Acinetobacter baumannii*^5,10^^.^. Cellulosic fabrics treated with CNPs offer enhanced antibacterial and colouring properties, as well as increased resistance to laundering, light, and scratching^11^. CNPs serve as an eco-friendly green anchor finish that strengthens the fabric's structure. Additionally, even after 10 washing cycles. The materials treated with CNPs possess antimicrobial properties against Gram-negative and Gram-positive bacteria^12^. CNPs have been proposed for usage as carriers of pharmaceuticals in cosmetics intended for skin and hair care. CNPs were utilised to deliver minoxidil sulfate (a hair growth medication) to hair follicles, providing a sustained release without skin exposure^13^. CNPs have been also utilised to deliver drugs through the ocular, oral, mucosal, buccal, nasal, pulmonary, or vaginal routes. Additionally, CNPs could be used as new therapeutic tools against various viral infections^1^. CNPs showed skin regenerative characteristics when tested on skin cell fibrocblasts and keratinocytes, establishing the foundation for anti-aging skin care products^14^. CNPs are appropriate nanomaterials for dental applications because of their ability to inhibit the formation of biofilm. Consequently, the application of CNPs in endodontics is attracting a lot of attention. del Carpio-Perochena et al.^15^ used CNPs instead of EDTA during root canal treatment. CNPs may also be used for food packaging as a filler in pectin-based edible films in order to improve the mechanical strength and barrier characteristics^16^.

CNPs are utilized in agriculture as eco-friendly and sustainable pesticides and fertilizers^17^, delivery of herbicides for weed removal^18^, in insecticide^19^, fungicide treatment^20^, and as carrier systems for gibberellic acid (plant growth hormone) application in sustainable agriculture^21^ as well as nano fertilizer to ensure the plants have a balanced nutrition of plants, enhances growth and crop yield^22^. Chitosan nanoparticles exhibit a higher capacity for wastewater treatment to remove a range of contaminants, including dyes, pesticides, and heavy metals, due to the presence of functional amino and hydroxyl groups in chitosan^23^.

The characteristics of CNPs vary greatly depending on the preparation and surface modification procedures that are applied ^7^. Different chemical and physical approaches have been developed to form CNPs including ionic gelation, spray drying, top-down approaches, precipitation-based methods, emulsification & crosslinking, etc.^17^. Chemical and physical approaches have numerous drawbacks, including the utilization of high-pressure, hazardous chemicals, temperature, energy, and the synthesized particles are large in size^17,24,25^. In previous literature, the average size of CNPs generated by self-assembly, nano spray dryer, and ionic gelation ranged from 166 to 3500 nm^22,26,27^ . Bekmukhametova et al.^28^ highlighted that, it is still challenging to develop protocol for the synthesis of CNPs ranging from 200 to 300 nm. Sharifi-Rad et al.^3^ reported that CNPs ranging in size from 10 to 80 nm have potential applications in the pharmaceutical, biomedical engineering and nanomedicine fields. Therefore, it is crucial to find safe strategies for the biofabrication of CNPs in order to generate ultrafine nanoparticles with a size below 100 nm, which is essential for numerous applications. Biofabrication of nanoparticles was accomplished with the help of microorganisms such as bacteria^29–32^, algal pigments^33,34^, algal derived soluble polysaccharides^35^ and fungi^36^. The biofabrication of CNPs is an eco-friendly process that yields ultrafine nanoparticles^4–7,37^. It has many advantages including biodegradability, nontoxicity, biocompatibility, and eco-friendliness^38^.

The present work describes a cost-effective, and eco-friendly technique for CNPs biofabrication using *Lavendula angustifolia* leaves extract. The biofabricated CNPs were characterized using UV–Visible spectroscopy, Transmission electron microscopy (TEM), FTIR (Fourier transform infrared spectroscopy), X-ray diffraction (XRD), Zeta potential and thermal characteristics analyses. As well as, face centered central composite design (FCCCD) was performed to optimize process of CNPs biofabrication. Finally, evaluation of their inhibitory activity against bacterial biofilm was performed.

In the present study, *Lavendula angustifolia* leaves extract was used to produce ultrafine CNPs with a size range between 7.24 and 9.77 nm. This is a crucial characteristic for many applications where the specific surface area is important.

## Materials and methods

### Preparation of plant leaves extract

Fresh *Lavendula angustifolia* leaves were gained from region of Alexandria, on the shores of the Mediterranean Sea and situated in the delta of the Nile of Egypt (at a latitude of 31.205753" and the longitude is 29.924526). The plant was kindly identified by Associate Prof. Maha El-Shamy, Botany Department, Faculty of Science, Mansoura University, Mansoura—Egypt. The voucher specimen (*Lavendula angustifolia*) has been deposited at the herbarium of Botany Department, Faculty of Science, Mansoura University, Mansoura—Egypt. “The *Lavendula angustifolia* leaves were collected, with permission, according to institutional, national, and international guidelines and legislation”. The leaves were thoroughly rinsed with tap water and then given a final rinse with distilled water to get rid of any impurities. Plant leaves extract was prepared according to the method of El-Naggar et al.^4^^.^. In a conical flask containing 100 mL of distilled water, 25 g of *Lavendula angustifolia* leaves were chopped into small pieces, immersed, agitated, and heated for 10 min. After boiling, the mixture was filtered through Whatman No. 1filter paper, and the filtered extract was collected and employed for CNPs biofabrication.

### CNPs biofabrication

Chitosan was purchased from Bio Basic Inc., Toronto, Canada, with a purity greater than ninety percent and a viscosity between 60 and 300). Chitosan with concentration at 1% (w/v) was dissolved in 1% acetic acid, kept under magnetic stirring for 24 h to ensure complete dissolving of chitosan in the solution. After dissolving, the pH was adjusted to 5 using 1N NaOH. Equal amounts from each of *Lavendula angustifolia* leaves extract and chitosan solution (1:1) were mixed and incubated at room temperature. After incubation, the turbidity that had generated was centrifuged at 10,000 × g for 10 min, washed, and then freeze-dried for further characterization.

### Characterization of CNPs

#### UV–Visible spectrum

To determine the maximum absorbance wavelength, the biosynthesized CNPs were analyzed by scanning them at a wavelength range between 200 and 400 nm using an Optizen Pop-UV/Vis spectrophotometer.

#### TEM investigations of CNPs samples

TEM (Transmission Electron Microscope) investigation, Energy Dispersive X-ray (EDX) spectroscopy analysis using carbon-coated copper grid for TEM and mapping analysis were performed using JEOL-JEM-2100 Plus, Ltd., Japan at SRTA- City, Alexandria, Egypt.

#### Zeta- potential of the synthesized CNPs

There is no reliable method for determining the surface charge of tiny particles in liquid. The zeta potential is a highly significant metric for determining the behavior of colloids or nanoparticles when they are suspended. Its value is highly correlated with particle surface shape and suspension stability. For this reason, it is used extensively in product stability investigations as well as surface adsorption research^39^. The ζ-potential of the biofabricated CNPs was quantified using a Malvern 3000 Zetasizer Nano ZS, UK at SRTA- City, Alexandria, Egypt.

#### Fourier transform infrared (FTIR) spectroscopy analysis

Sample of chitosan, CNPs and lypholized *Lavendula angustifolia* leaves extract were ground with KBr pellets and utilised for FTIR analysis. Shimadzu FTIR-8400 S spectrophotometer was used to analyze the surface characteristics of the biofabricated CNPs in the range of 4500–500 cm^−1^.

#### XRD pattern

The crystallinity of the CNPs and structural properties were determined by XRD using advanced X-ray diffractometer (Bruker D2 Phaser 2nd Gen) equipped with a CuKα radiation, λ = 1.5406 A°, current 30 mA, applied voltage 10 kV. 2θ between 10 and 60 and the scanning rate was 2°/min.

#### CNPs' thermal characteristics

Thermogravimetric analysis (TGA) was conducted on CNPs sample using a TGA-50H thermogravimetric analyzer of type 50-H. CNPs sample weighing 6 milligrammes was exposed to temperatures ranging from ambient temperature to 800 °C at an increment rate of 10 °C min^−1^. The flow rate used was 40 mL/min. Using Differential Scanning Calorimetry (DSC) analysis, the pyrolysis pattern of CNPs was investigated. CNPs sample weighing 3.2 milligrammes was analyzed under nitrogen atmosphere conditions. The scan temperatures range from room temperature to 300 °C.The flow rate used was 30 mL/min.

### Optimization of CNPs using face-centered central composite design (FCCCD)

The optimum levels of three independent variables and their effects on CNPs biofabrication were determined using FCCCD. The three variables vary on three different levels The tested independent variables were chitosan concentration (X_1_; 0.5, 1, 1.5%), concentration of leaves extract (X_2_; 50, 75, 100%), and initial pH level (X_3_; 4, 4.5, 5). Twenty experimental runs, of which 6 were performed in the middle levels of the experiment. The use of the polynomial equation of the second degree allowed for the determination of the interactions that existed between the process independent variables and the CNPs biofabrication.

1 $$\varvec{Y = \beta }_{\varvec{0}} \varvec{ + }\sum\limits_{\varvec{i}} {\varvec{\beta }_{\varvec{i}} \varvec{X}_{\varvec{i}} } \varvec{ + }\sum\limits_{{\varvec{ii}}} {\varvec{\beta }_{{\varvec{ii}}} \varvec{X}_{\varvec{i}}^{\varvec{2}} } \varvec{ + }\sum\limits_{{\varvec{ij}}} {\varvec{\beta }_{\varvec{i}} \varvec{X}_{\varvec{i}} \varvec{X}_{\varvec{j}} }$$

In which Y is the predicted CNPs biofabrication (mg/mL), X_i_ is the coded values of the independent factors, the linear coefficient (β_i_), β_0_ represents the regression coefficients, β_ij_ is the interaction coefficients and quadratic coefficients (β_ii_).

### Statistical analysis

For both the designing of the experiments and the carrying out of the statistical analysis, the programme Design Expert version 12 for Windows was employed (https://www.statease.com/software/design-expert/). The STATISTICA programme, version 8.0 (StatSoft Inc., Tulsa, USA) was used for plotting the three-dimensional surface plots (https://www.statsoft.de/de/software/statistica). The artificial neural network (ANN) analysis was performed using JMP pro version 16.2 (JMP, SAS Institute Inc., Cary, NC) (https://www.jmp.com/en_in/home.html).

### Antibiofilm activity of CNPs

The ability of CNPs, in different doses (10, 20, 50, 100, 200, 500, 1000, 1500 μg/mL), to prevent biofilm-forming pathogens (*P. aeruginosa* (ATCC 27,853), *S. aureus* (ATCC 25,923) and *C. albicans* (ATCC 10,231) from the adherence to a polystyrene surface of microtiter plate was evaluated. Via microdilution assay, each well in sterile tissue culture microtiter plate with U-bottomed was dispended with 100 μl of sterile Trypticase Soy Broth (TSB) supplemented with 1% w/v glucose, inoculated with 20 μl of pre-adjusted culture (1 × 10^6^ CFU/mL) of examined pathogens. Besides, set of positive and negative controls were examined in parallel. Wherein, the wells containing CNPs-free medium and culture-free medium were deemed as positive controls and negative controls, respectively. The extract of *Lavendula angustifolia* was also examined in parallel. After incubation at 37 °C for 24 h under static conditions, the mature biofilms were washed, fixed, stained with (0.1%, w/v) crystal violet and quantified spectrophotometrically at 595 nm by ELISA reader (Tecan Infinite M200, Switzerland) as described in details by Elyamny et al.^40^. The antibiofilm activity of CNPs was calculated as represented in the following equation

2 $${\text{Biofilm inhibition }}\% = \left( {A - A_{o} } \right) \, / \, A \, * \, 100$$

where A and A_0_ pointed out to the absorbance of the positive control and the treated wells, respectively.

### Impact of CNPs on biofilm metabolism and biochemical constituents

To measure the metabolic activity of live cells adhered in the biomass of biofilm, MTT colorimetric method was employed. The overnight biofilm was prepared exactly as described in “antibiofilm activity assay”. Immediately after completion of incubation period, the planktonic content of each well was removed and rinsed three successive times with distilled water. About 150 μL of 0.25 mg/mL MTT solution (3-[4, 5- dimethylthiazol-2-yl]-2, 5-diphenyltetrazolium bromide was mixed thoroughly with the adhered cells within biofilm matrix. The microtiter plate was incubated at 37 °C for 2–3 h. Thereafter, the solution was decanted and 2% DMSO was blended evenly to dissolve insoluble purple formazan. Finally, the intensity of active live cells was determined by detecting the absorbance at 570 nm by using microtiter ELISA reader. Higher absorbance indicates higher number of active surviving cells in the biofilm. The inhibition percentage was calculated following the previous equation (Eq. 2)^41^.

To define the influence of different doses of CNPs on biofilm components (i.e., crude extracellular polymeric substance (EPS) and protein), the microtiter plate was prepared, inoculated incubated, processed and washed as described in section “antibiofilm activity assay”. The adhered biofilms matrix was dislodged through vigorous pipetting with pipet tips and suspended in phosphate-buffer saline (PBS, pH 7.2 ± 0.2). The obtained supernatants of the adherent biofilm were used to measure the protein and carbohydrate yield via Bradford and phenol–sulfuric assays, using BSA and glucose as standards, respectively; following methods described deeply by Shawki et al.^41^.

### Ultrastructure study of biofilms upon CNPs treatment

The morphological changes of biofilm architectures induced by lethal dose of CNPs were visualized by scanning electron microscope (SEM). In a 12-well polystyrene microtiter plate, biofilms were developed on sterile glass coverslips (9 mm) immersed in wells inoculated with 10^6^ CFU/mL in the control and CNPs-treated. After incubation, the coverslips were gently removed using sterile tweezers and washed with 0.85% NaCl to remove planktonic cells. The treated and untreated samples were prefixed in 2.5% glutaraldehyde for 24 h at 4 °C and dehydrated with gradient ethyl alcohol series (30:100%) for 15 min. The dried samples were subjected to a Polaron SC7620 Sputter Coater for gold coating step and inspected using SEM (JEOL JSM 6360LA, Japan)^42^.

### Data analysis

All tests were implemented in triplicate, the data were expressed as means ± SEM (Standard Error of Mean). The Graphpad Instat software was used for statistical analyses by one-way ANOVA with Tukey’s post hoc. Statistical significance was considered at the *P*-value ≤ 0.05.

## Results and discussion

The production of CNPs has been performed using a very diverse range of approaches. When choosing an appropriate process for the preparation of CNPs, it is important to consider the stability and safety of the CNPs as well as their particle size. The biofabrication of CNPs provides several advantages, such as being a one-step process that is environmentally friendly, non-toxic, and energy-efficient. Furthermore, biosynthesized CNPs are more stable^6^. CNPs were biosynthesized in a green manner with the use of microorganisms such as actinomycetes^6^ and fungi^37^. Moreover, secondary metabolites present in aqueous extracts of plant leaves were used as reducing agents in the nanoparticles biofabrication^4,5^. Biomolecules can act as reducing and capping agents^43^. Chandran et al.^44^ also stated that the biological molecules can act as either stabilisers or reducers, or perhaps both of these roles, during the biofabrication process of nanoparticles. In the present investigation, a strategy for the cost-effective, eco-friendly, and biosafe synthesis of nanoparticles by using *Lavandula angustifolia* (lavender) leaves extract was used.

**Figure 1:**
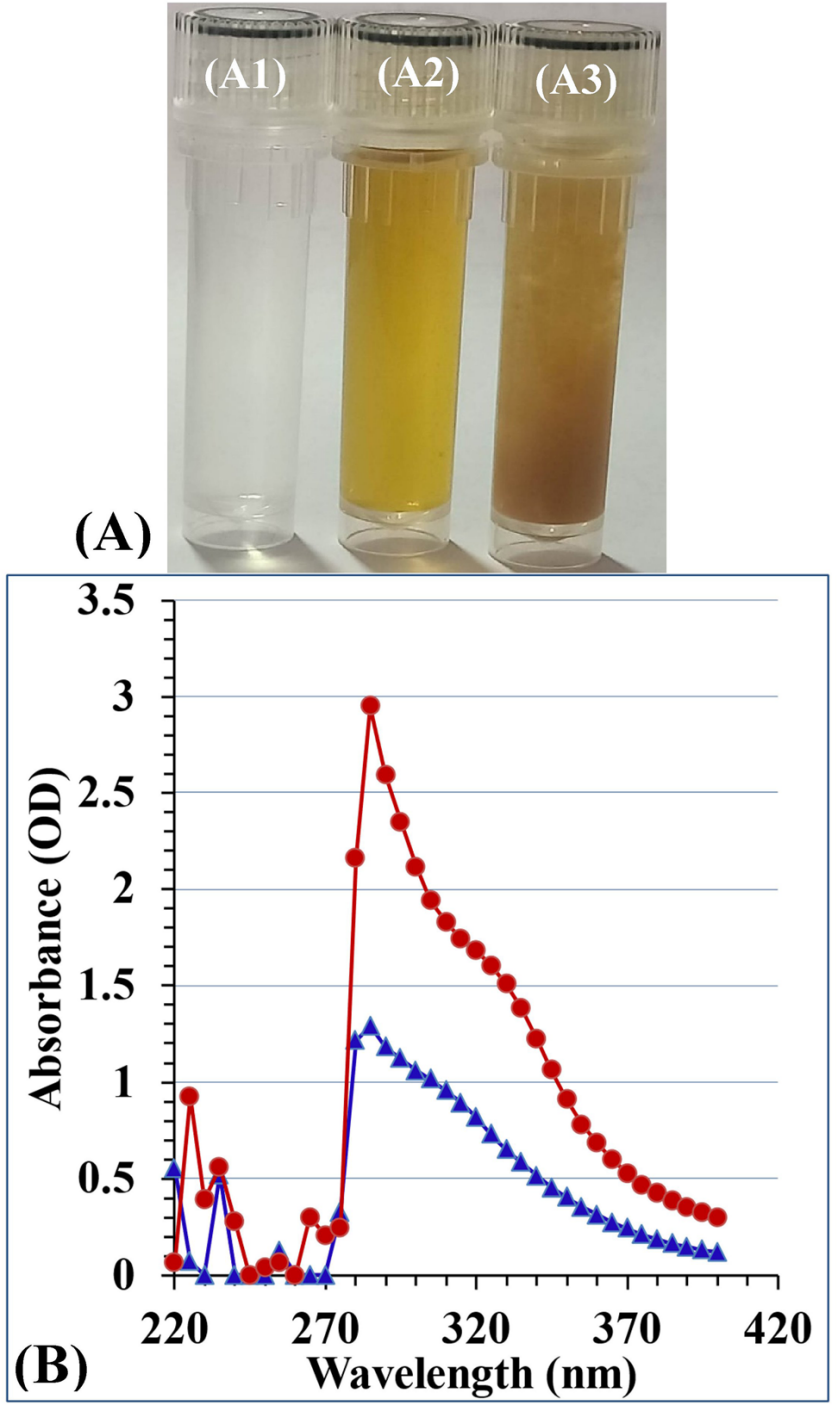
(**A**) Vial of chitosan solution (A1), *Lavendula angustifolia* leaves extract (A2), and the biosynthesized chitosan nanoparticles (A3 using *Lavendula angustifolia* leaves extract, (**B**) UV/visible spectra of chitosan (blue line) and chitosan nanoparticles (red line)(the maximum absorbance wavelength of CNPs at 285 nm).

Lavender is a strongly aromatic shrub of the Lamiaceae family and is native to the Mediterranean region. It is commonly known as *Lavandula officinalis*^45^. A phytochemical analysis of *Lavandula angustifolia* aqueous extract showed the presence of condensed tannins, flavonoids and phenolic contents. In addition, essential oil was found to include four major constituents: α-Campholenal, ρ-cymene, Linalyl acetate, and Linalool^46^. Lavender contains tannins, coumarin, herniarin, ursolic acid, valeric acid, glycolic acid, coumaric acid, minerals, sugars, phytosterols, anthocyanins, and essential oil^47^. Accordingly, *Lavendula angustifolia* leaves extract can act as an eco-friendly, cost-effective, and biosafe reductant for chitosan molecules and their corresponding chitosan nanoparticles. Figure 1 shows a vial of chitosan solution (A1), *Lavendula angustifolia* leaves extract (A2), and the biosynthesized chitosan nanoparticles (A3) using *Lavendula angustifolia* leaves extract.

### UV/visible spectrum of CNPs biosynthesized using *Lavendula angustifolia* leaves extract

To identify the absorbance peak of the biologically synthesised CNPs, a UV/Vis spectrophotometer scan was conducted over the wavelength range of 220 to 400 nm. Figure 1B shows UV/visible scan spectrum of chitosan and chitosan nanoparticles. Chitosan nanoparticles displayed a distinct single peak with the maximum absorbance wavelength at 285 nm (Fig. 1B). It was reported that the UV–visible spectrum of chitosan nanoparticles ranged between 200 and 322 nm as a result of the presence of the CO group^48^. The present findings are also in agreement with those of Sathiyabama and Parthasarathy^37^, who used proteins derived from *Penicillium oxalicum* to produce chitosan nanoparticles with a sharp peak at 285 nm. On the other hand, it was reported that CNPs biosynthesized using *Pelargonium graveolens* leaves extract displayed a maximum absorbance peak wavelength at 295 nm^4^. Also, CNPs biosynthesized by the aqueous extract of *Eucalyptus globulus* leaves extract as a bioreductant displayed the highest absorbance at 295 nm^5^.

### Transmission electron microscope (TEM) examination

**Figure 2:**
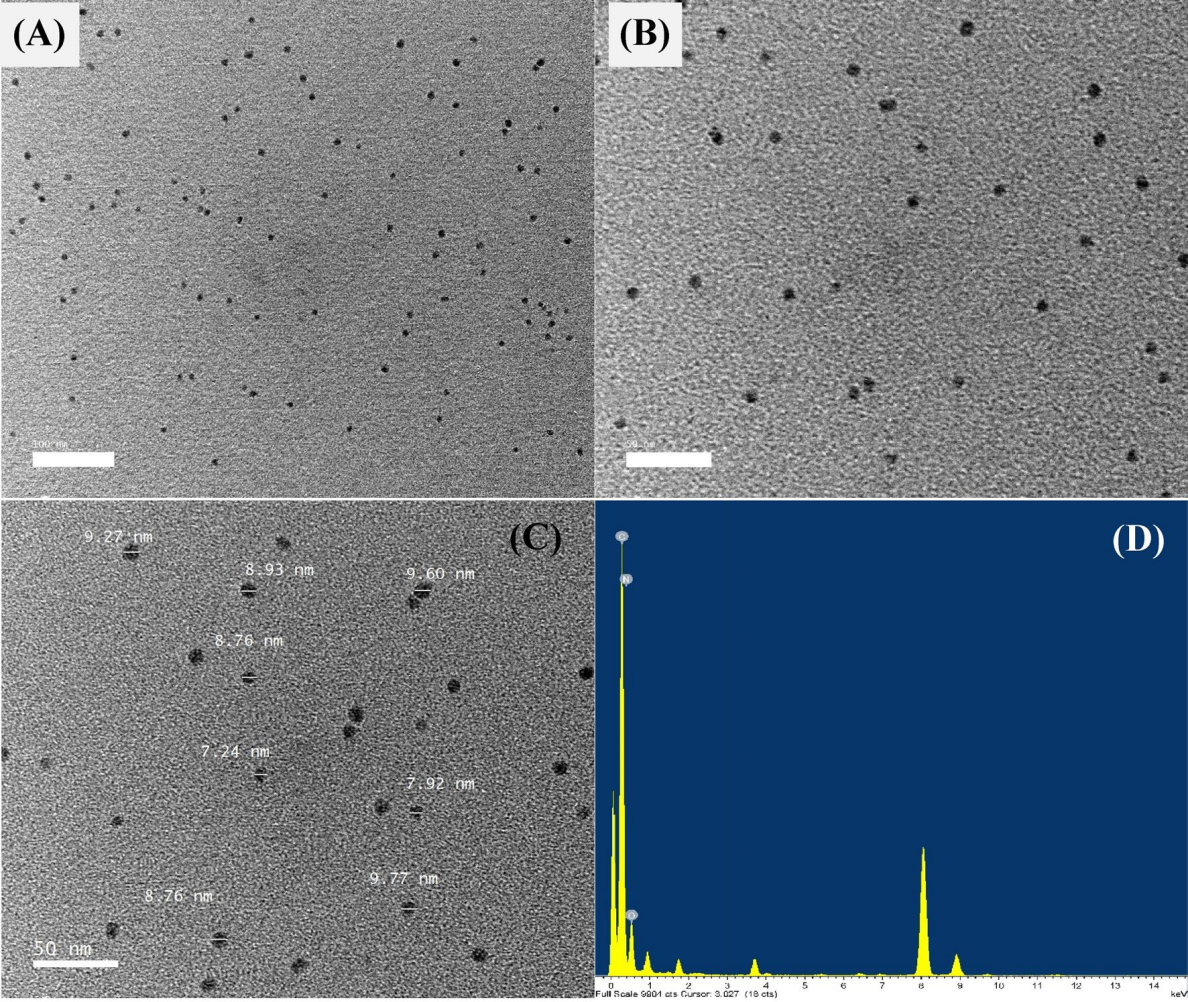
The biosynthesized chitosan nanoparticles using *Lavendula angustifolia* leaves extract, as detected by the micrographs of TEM (**A**–**C**). Analyses of chitosan nanoparticles bio-synthesized using *Lavendula angustifolia* leaves extract with EDX (**C**).

TEM has been used for many years to investigate the shape, size, composition, dispersion, and aggregation of the explored material's nanoparticles. The biozyntheized CNPs using *Lavendula angustifolia* leaves extract were subjected to morphological characterization using TEM. TEM examination revealed that the biosynthesized CNPs were irregular in shape, and their sizes ranged from 7.24 to 9.77 nm with no evidence of agglomeration. The TEM image also showed that the chitosan nanoparticles surface was characterized by a relatively coarse texture (Fig. 2A, B & C). According to El‑Naggar et al.^4^, the CNPs biosynthesized by *Pelargonium graveolens* were spherical and exhibited excellent dispersion. The smaller particle size of CNPs improves drug delivery and, consequently, efficacy, as they are easier to transfer through biological membranes^49^. A smaller particle size offers the potential to encapsulate more pharmaceutical ingredients, enhance the drug's stability and absorption, and allow for longer administration times^49^.

Van et al.^50^ stated that the size distribution of CNPs synthesized by nano spray dryer ranged from 300 to 3500 nm , with an average size of approximately 1000 nm, depending on the hole diameter of spray caps. According to the findings of Nguyen et al.^27^, the molecular weight of chitosan and the size of the spray dryer nozzles both had an effect on the average size of the CNPs, which was found to be between 166 and 1230 nm. Ha et al.^22^ found that the size distribution of the CNPs produced by ionic gelation of chitosan solution and tripolyphosphate (TPP) varied between 300 and 750 nm. In Agarwal et al.^51^ study, the size of CNPs produced by chitosan and TPP varied between 168 and 682 nm. In addition, the size of the self-assembled CNPs ranged from 277 to 731 nm^26^.

**Figure 3:**
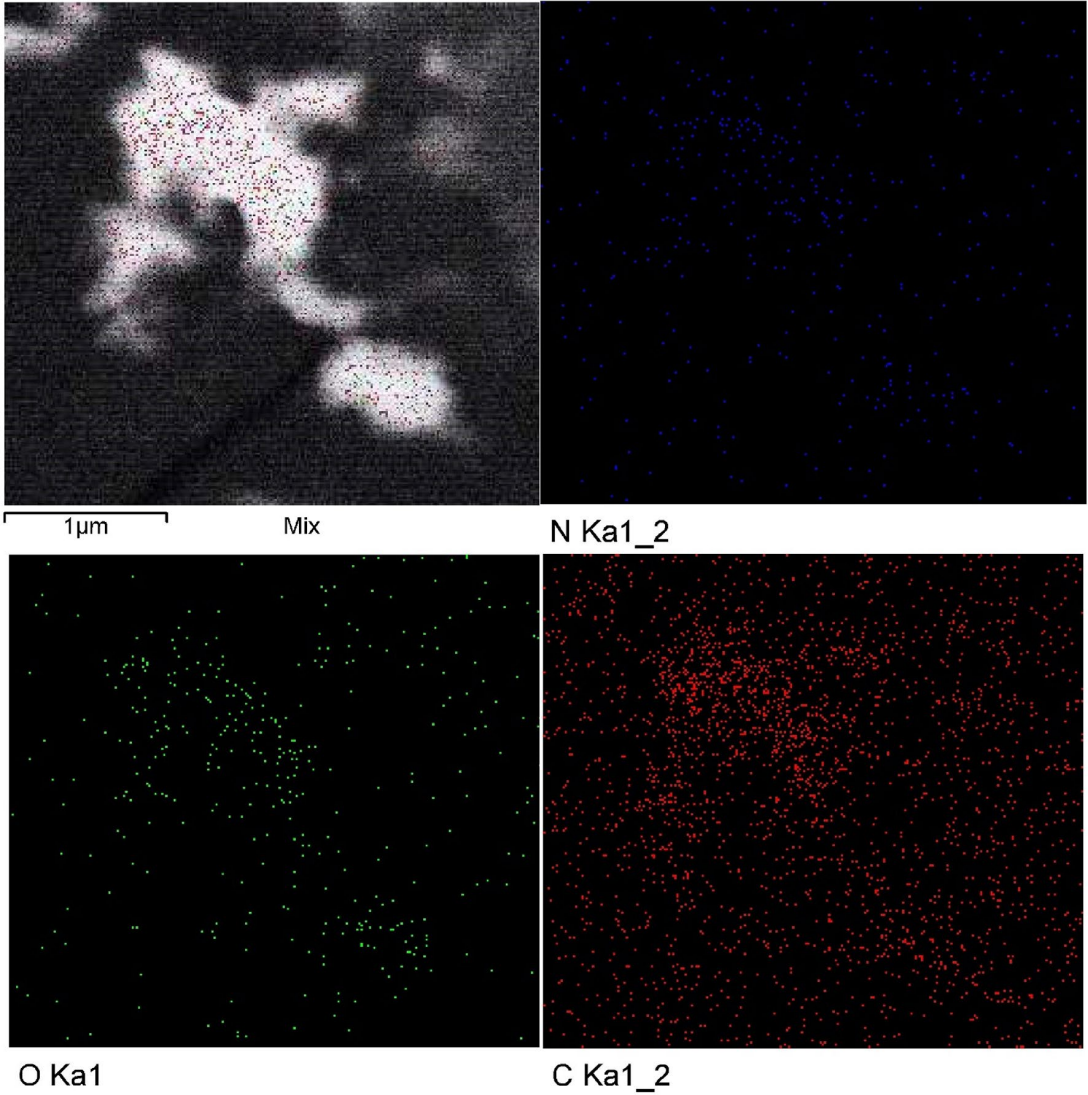
Mapping analysis of the bio-synthesized using *Lavendula angustifolia* leaves extract.

Energy-dispersive X-ray spectroscopy (EDX) analysis was used to investigate the chemical composition and principal constituents of boisynthesized CNPs. Figure 2D shows the EDX analysis of CNPs synthesized *by Lavendula angustifolia* leaves extract. EDX analysis revealed that the obtained CNPs contain nitrogen, oxygen and carbon. In order to explore the pattern of biosynthesized CNPs distribution by *Lavendula angustifolia* leaves extract, mapping analysis of CNPs was carried out.TEM elemental mapping reveals that CNPs and their constituents (oxygen, nitrogen, and carbon) are uniformly dispersed and distributed (Fig. 3).

### Fourier transform infrared (FTIR) analysis

**Figure 4:**
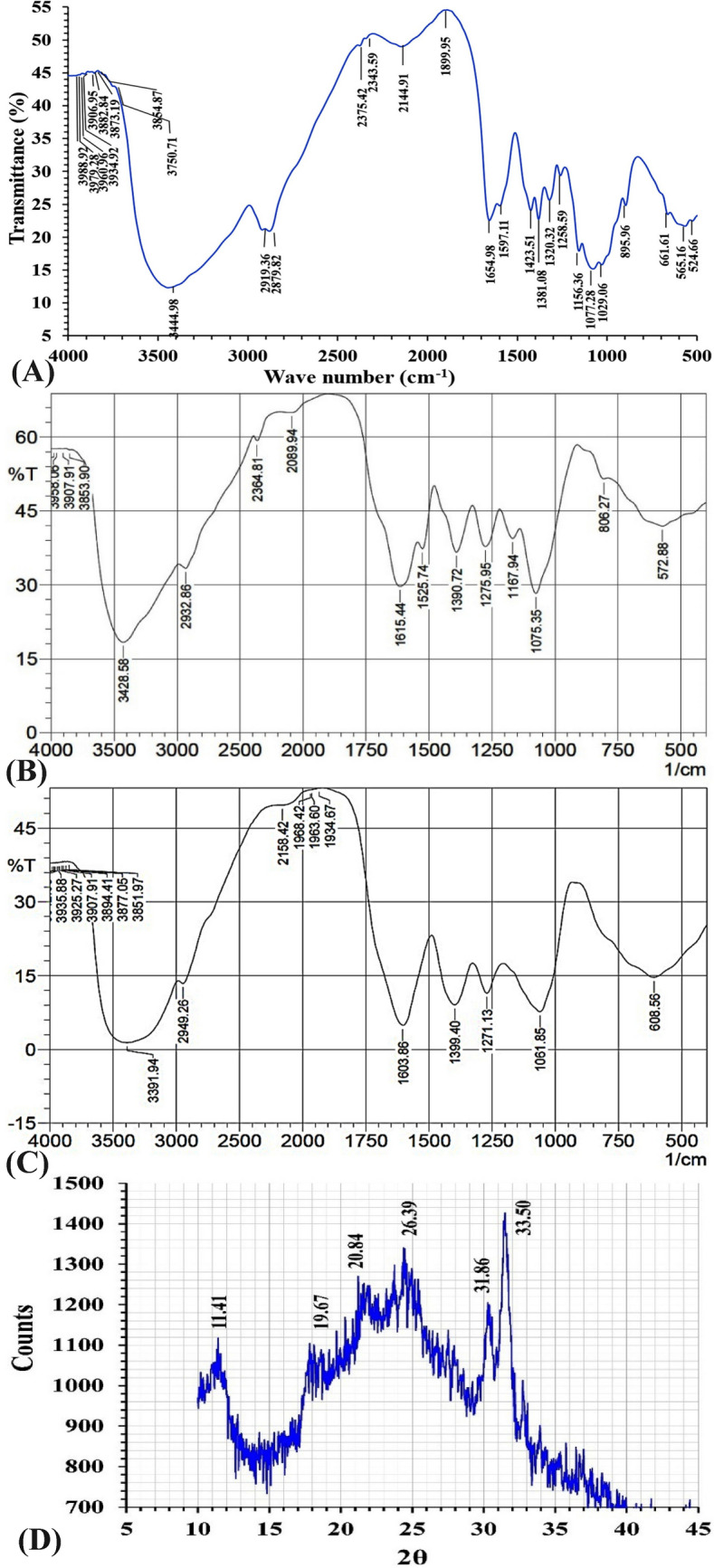
Examination of FTIR analyses for chitosan standard (**A**), FTIR for the bio-synthesized chitosan nanoparticles (**B**), FTIR for *Lavendula angustifolia* leaves extract (**C**) and XRD (**D**) of CNPs bio-synthesized using *Lavendula angustifolia* leaves extract.

FT–IR analysis was conducted for characterization and identification of the functional groups found in the biosynthesized CNPs using *Lavendula angustifolia* leaves extract. FTIR spectrum of the biosynthesized CNPs was analyzed and compared with the FTIR spectrum of a chitosan standard (Fig. 4A, B). The first group of bands appeared in the spectra between 4057 and 3750 cm^−1^, indicating the combination of functional groups of –NH_2_,–CH, C– C, and –OH. Significant shifts of peaks in the spectrum of CNPs from peaks in the spectrum of the chitosan standard indicate a significant role of functional groups in the CNPs biofabrication. The presence of a broad band around 3444.98 cm^−1^ in chitosan standard sample due to the stretching vibration of O–H and N–H^52^. Moreover, on the formation of nanoparticles, the stretching vibration of O–H and N–H groups at wavelength 3444.98 cm^−1^ found in the spectrum of chitosan standard shifted to 3428.58 cm^−1^ in the CNPs spectrum which indicates the stretching vibrations of OH groups. A characteristic peak at 3428.58 cm^−1^ appeared in the spectrum of CNPs can be attributed to –NH_2_ and –OH groups stretching vibration^53^. The C–H stretching vibration of the polymer backbone of chitosan is indicated by peak at 2919 cm^−1^^54^ shifted in the CNPs spectrum to 2932 cm^−1^ which indicates the stretching vibrations of CH alkanes^55^.The characteristic peak at 1654.98 cm^−1^ in the spectrum of the chitosan standard indicated the vibrations of carbonyl group (amide band I)^56^. In CNPs spectrum, this peak is sharper and shift at 1615.44 cm^−1^ (amide I, β-sheet)^57^ indicating interactions between protonated amine groups of the chitosan standard with the components of *Lavendula angustifolia* leaves extract. The formation of CNPs is indicated by the shift of vibrations from higher to lower wave number^58^. The characteristic absorption band at 1381 cm^−1^ in FTIR spectrum of chitosan standard indicated the vibrations of Amide III region^58^. In CNPs spectrum, this peak is sharper and shift at 1390 cm^−1^ that could be assigned to CH_3_ of the amide group^59^. In addition, in FTIR spectrum of chitosan, the peak at 895 cm^−1^ is attributed to stretching vibration of of saccharide moiety (C–O–C)^60^. This peak is shifted in the CNPs spectrum to 806 cm^−1^ which belongs to CH ring-wagging vibration^61^. After CNPs production, the peaks in the FTIR spectrum at 1423, 1320, 1029, 661, and 524 cm^−1^ disappeared, showing that these groups are involved in the CNPs biofabrication with the components of *Lavendula angustifolia* leaves extract. FTIR analysis has been also performed for lypholized *Lavendula angustifolia* leaves extract as a control (Fig. 4C). The presence of capping groups on the surface of CNPs is confirmed by FTIR analysis. These groups serve to stabilize the CNPs and prevent any agglomeration or aggregation that may occur in the colloidal phase.

### X-ray diffraction (XRD)

XRD examination is helpful in defining the crystalline structure of samples in terms of their physical properties. In this study, XRD was applied for examination of CNPs in terms of crystalline structure. The XRD of the current biosynthesized CNPs by *Lavendula angustifolia* leaves extract showed sex peaks at 2*θ* of 11.41, 19.67, 20.84, 26.39, 31.86 and 33.5° (Fig. 4D) indicating a shift from the normal chitosan peaks. The peaks of chitosan appeared at 2*θ* within the range of (20-30º) and it has a hump peak because of its amorphous structure^62^. The XRD patterns of chitosan showed three strongest distinctive peaks located at 2θ = 20.4, 26.4 and 29.5°^63^. According to Rasaee et al.^64^, the CNPs displayed diffraction peaks at at 2θ = 10° and 20°. These peaks demonstrated that the chitosan possessed a high degree of crystallinity. The crystalline peaks at 19.67, 20.84 could be ascribed to the shift of crystalline peaks of chitosan.

### CNPs' thermal characteristics

**Figure 5:**
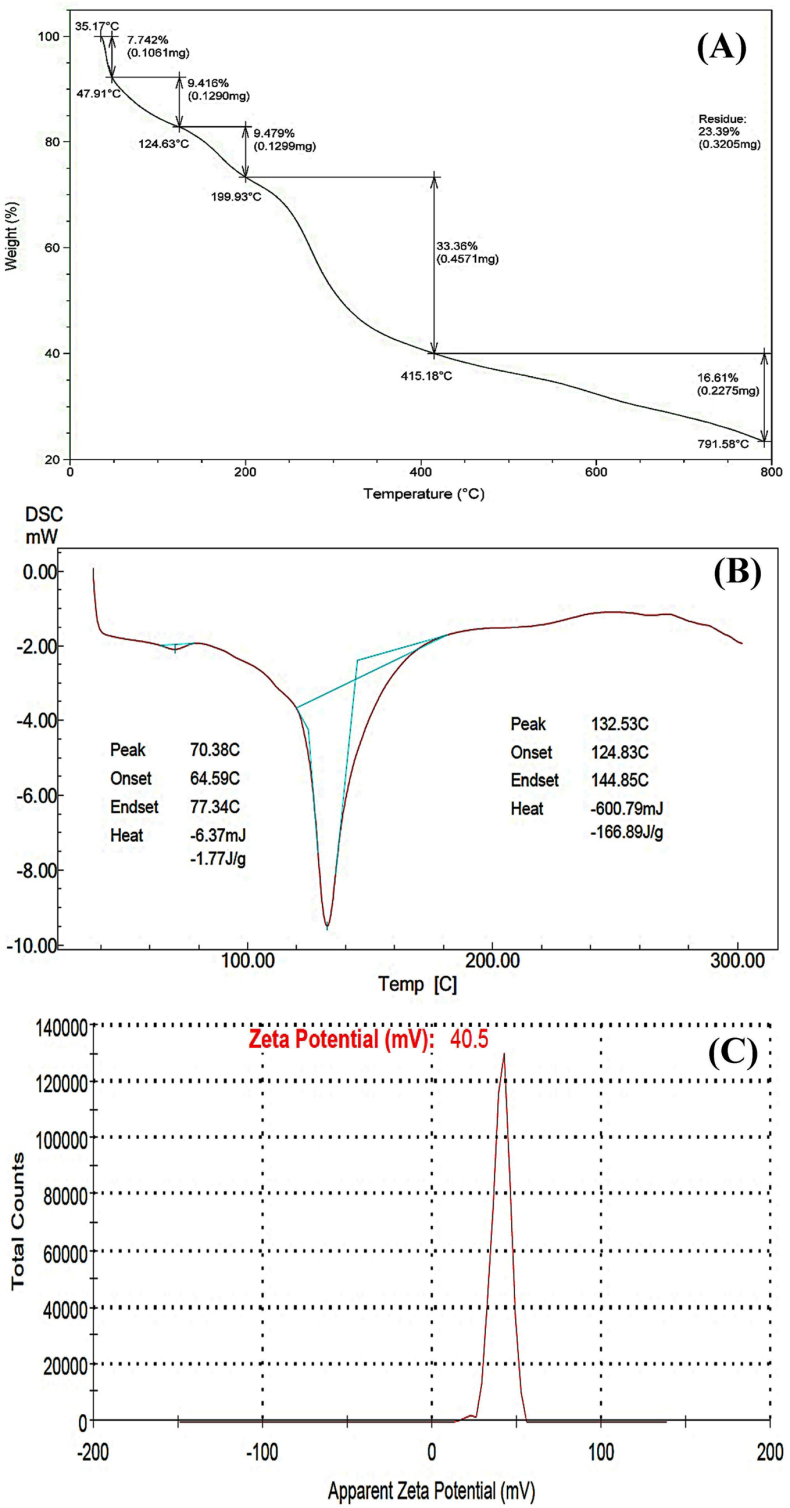
Chromatograms of SDT (**A**), DSC (**B**) and (**D**) ζ-potential investigation of CNPs bio-synthesized using *Lavendula angustifolia* leaves extract.

TGA and DSC were the basic techniques that were used in order to determine the thermal properties of the biosynthesized CNPs using *Lavendula angustifolia* leaves extract. The thermal behavior of CNPs was performed by TGA to investigate mass variation, the heating ratio is constantly changing (between room temperature and 800 °C). This technique is typically used to examine the effects of varying heating rates on the nanoparticles being tested. The changes in temperature reflected the changes of nanopartilces mass which is represented in Fig. 5A.

Figure 5A showed that a rapid initial mass reduction (-7.74%) can easily be detected when raising the temperature from 35.17 up to 47.91 °C, that represent 0.1061 mg due to breakdown of volatile units as reported by Vijayalakshmi et al.^65^ or dehydration of the saccharide rings process that does not involve chemical reactions or structural changes^66^. With increasing temperatures, the weight loss of biologically synthesized CNPs exhibited a multistage breakdown. The highest weight loss (33.36%; 0.4571 mg) was seen at a temperature between 199.93 and 415.18 °C due to chitosan thermal degradation^66^. At 791.58 °C, approximately 83.39% of the CNPs sample gets destroyed, leaving 16.61% of the sample as leftover, demonstrating higher thermal stability. Increased thermal stability suggests that crosslinking has caused the hydrogel network to become stronger and more rigid^65^. Although TGA can help, it may not be enough to find products that have been damaged. Therefore, besides TGA, DSC is crucial for identifying the existence of intermediate degradation products^67^.

Differential scanning calorimetry (DSC) is a technique for measuring the thermal effects of phase transitions and chemical processes as a function of temperature. To demonstrate the degree of variation in the CNPs heat flow as a result of temperature, the DSC analysis was carried out at various rates of heating (Fig. 5B). Two distinct endothermic peaks appeared as a result of the thermal effects of the alteration. The broad endothermic peak was observed at 70.38 °C between 64.59 and 77.34 °C, requiring a heat amount of − 1.77 J/g CNPs. The second endothermic peak appeared at 132.53 °C was seen between 124.83 and 144.85 °C, require an amount of heat equivalent to − 166.89 J/g. In comparison with the TGA, the DSC does not measure the loss of particle weight; instead, it measures the heat adsorption. The biosynthesized CNPs using *Lavendula angustifolia* leaves extract react with the heat exothermic process, meaning that the particles were formed in a crystallization manner. Mohammed et al.^68^ reported that the exothermed particles were characterized by purity, crystallinity, and stability. The crystals of the chitosan nanoparticles did not accept heat flow above 130 °C, this result indicates that the biosynthesized chitosan nanoparticles contain pure crystals.

### Zeta (ζ) potential analysis

The surface effects are greatly influenced by the dispersion of nanoparticles. Agglomeration of nanoparticles is caused by the strong attractive interactions between particles, which reduce their surface area and, consequently, their nanoscale characteristics. It's well known that the electronic repulsion among the examined particles can significantly affect the particles' stabilization; for that reason, the higher the zeta potential value refelected, the higher the particle stability. The zeta potential of a particle is a parameter that represents the particle's charge and indicates the particle's stability.

In general, suspended particles will not aggregate if they all have a large zeta potential (either negative or positive), which will act as a repulsive force between them. In contrast, there is no force preventing particles with a low zeta potential from aggregating and flocculating. Zeta potential value is crucial for understanding and predicting particle interactions in suspension (Wangoo et al., 2010)^69^.

The ζ-potential (Fig. 5C) displays a single peak, indicating the homogeneity and stability of biosynthesized CNPs produced by *Lavendula angustifolia* leaves extract, which were positively charged with ζ-potential value of + 40.5 mV. Kheiri et al.^70^ revealed that the zeta potentials of CNPs were positive due to the presence of residual protonated amine groups. Despite the physical stability of the suspension, a zeta potential of at least ± 30 mV is required for an NPs suspension to be stabilized by electrostatic repulsion^71^. CNPs are less stable because of reduced electrostatic repulsion if their zeta potential is less than + 30 mV^24^. According to the findings of Qi et al.^72^, CNPs have a positive charge of about 51 mV on their surfaces. Khan et al.^73^, Raza & Anwar^74^ and Asal et al.^75^ all report that the surface zeta potential of CNPs was measured to be + 31, + 31.3 and + 31 mV; respectively. Zeta potential was in the range of 13.2–42.5 mV^76^. On the other hand , the zeta potential ranging between 21.8 mV and 26.3 mV for the examined particles meant that these particles are unstable colloidal particles that possess a certain tendency to agglomerate. This could be due to the exictence of residual amine groups^77^.

### Statistical optimization of chitosan nanoparticles biofabrication using *Lavendula angustifolia* leaves extract using face centered central composite design (FCCCD)

The yield of biosynthesized CNPs is influenced by many independent factors such as temperature, initial pH level, incubation time, and chitosan concentration^7^. The impacts of three factors on CNPs biofabrication (as a response) were investigated in the present study. These three variables were the initial pH level, chitosan concentration, and leaves extract concentration. The bioprocess variables of CNPs biofabrication were optimized with the help of the FCCCD in order to maximize CNPs biofabrication and investigate the individual, interaction, and quadratic effects of process variables on CNPs biofabrication using *Lavendula angustifolia* leaves extract. Twenty FCCCD experiments were conducted to identify the optimal values for the variables of interest. The design matrix in Table 1 shows the main studied variables, their actual and coded levels, the experimental and predicted biosynthesized CNPs values (mg/mL), and their residual values. To calculate the experimental errors, six experiments were conducted in the central sites.

**Table 1:** FCCCD matrix mean actual and predicted values of chitosan nanoparticles biofabrication using *Lavendula angustifolia* leaves extract.

Std	Run	Type	X_1_	X_2_	X_3_	Actual	Chitosan nanoparticles biofabrication (mg/mL)				
							FCCCD		ANN		
							Predicted	Residuals	Predicted	Residuals	Validation
14	1	Axial	0	0	1	7.63	7.31	0.32	7.59	0.04	Validation
13	2	Axial	0	0	− 1	0.58	0.80	− 0.22	0.53	0.05	Validation
12	3	Axial	0	1	0	3.76	3.76	− 0.01	3.75	0.01	Training
15	4	Center	0	0	0	4.17	4.03	0.15	4.06	0.11	Training
8	5	Factorial	1	1	1	6.75	6.92	− 0.17	6.89	− 0.14	Validation
1	6	Factorial	− 1	− 1	− 1	1.37	1.22	0.15	1.37	0.00	Training
17	7	Center	0	0	0	4.02	4.03	0.00	4.06	− 0.04	Training
19	8	Center	0	0	0	4.01	4.03	− 0.01	4.06	− 0.05	Training
11	9	Axial	0	− 1	0	5.56	5.46	0.10	5.57	− 0.01	Training
7	10	Factorial	− 1	1	1	6.28	6.24	0.04	6.28	0.00	Training
2	11	Factorial	1	− 1	− 1	2.26	2.33	− 0.06	2.26	0.00	Training
20	12	Center	0	0	0	4.10	4.03	0.07	4.06	0.04	Validation
5	13	Factorial	− 1	− 1	1	7.93	8.18	− 0.25	7.93	0.00	Training
10	14	Axial	1	0	0	4.33	4.38	− 0.05	4.33	0.00	Training
9	15	Axial	− 1	0	0	3.63	3.49	0.14	3.68	− 0.05	Validation
18	16	Center	0	0	0	3.79	4.03	− 0.24	4.06	− 0.27	Validation
4	17	Factorial	1	1	− 1	1.09	0.87	0.22	1.09	0.00	Training
16	18	Center	0	0	0	3.87	4.03	− 0.16	4.06	− 0.19	Validation
6	19	Factorial	1	− 1	1	9.93	9.87	0.06	9.93	0.00	Training
3	20	Factorial	− 1	1	− 1	0.69	0.77	− 0.08	0.69	0.00	Training

Variable	Variable code	− 1	0	1							
Initial pH level	X_1_	4	4.5	5							
Chitosan conc. (%, w/v)	X_2_	0.5	1	1.5							
Leaves extract conc. (%, v/v)	X_3_	25	50	75							

Based on the variance of the three factors, FCCCD experiments for biosynthesized CNPs using *Lavendula angustifolia* leaves extract indicate significant variability. Measured data indicates that concentrations of biosynthesized CNPs varied from 0.58 to 9.93 mg/mL. Run 19 with a pH of 5, 0.5% chitosan concentration, and 75% leaves extract concentration produced the greatest concentration of biosynthesized CNPs (9.93 mg/mL). In contrast, run 2 (pH 4.5, 1% chitosan, 25% leaves extract) produced the lowest concentration of biosynthesized CNPs (0.58 mg/mL).

### Multiple regression analysis and analysis of variance (ANOVA)

The FCCCD results for the biosynthesized CNPs using *Lavendula angustifolia* leaves extract were analysed statistically using multiple regression analysis and analysis of variance (ANOVA). To determine the model's reliability, the coefficient estimates values, R^2^ value, predicted R^2^ value, adj R^2^ value, *P*-value (probability value), lack of fit, and *F*-value (Fisher value) shown in Table 2 were calculated and evaluated. The linear, interaction, and quadratic effects of the three process factors of interest were also investigated^78^.

**Table 2:** Analysis of variance for chitosan nanoparticles biofabrication using *Lavendula angustifolia* leaves extract obtained by FCCCD.

Source of variance		Sum of squares	*df*	Mean square	*F-*value	*P-*value *P*rob > *F*	Coefficient estimate
Model		118.36	9	13.15	281.61	< 0.0001	4.03
Linear effects	X_1_	2.00	1	2.00	42.73	< 0.0001	0.45
	X_2_	7.19	1	7.19	153.95	< 0.0001	− 0.85
	X_3_	105.87	1	105.87	2267.11	< 0.0001	3.25
Interaction effects	X_1_ X_2_	0.51	1	0.51	10.86	0.0081	− 0.25
	X_1_ X_3_	0.17	1	0.17	3.61	0.0866	0.15
	X_2_ X_3_	1.10	1	1.10	23.56	0.0007	− 0.37
Quadratic effects	X_1_^2^	0.02	1	0.02	0.51	0.4923	− 0.09
	X_2_^2^	0.94	1	0.94	20.14	0.0012	0.58
	X_3_^2^	0.00	1	0.00	0.06	0.8112	0.03
Error effects	Lack of Fit	0.3658	5	0.0732	3.62	0.0923	
	Pure Error	0.1012	5	0.0202			

Std. Dev	0.22	R^2^	0.9961				
Mean	4.29	Adj R^2^	0.9925				
C.V. %	5.04	Pred R^2^	0.9581				
PRESS	4.97	Adeq Precision	59.53				

* Significant values, *F*: Fishers's function, *P*: Level of significance, C.V: Coefficient of variation.

The coefficient of determination (R^2^) value for the model that is currently being used is 0.9961. When the model had an (R^2^) value that was greater than 0.9, it was regarded as having a high degree of correlation^33^. In the current study, we found that the R^2^ value of the model that was applied to the biosynthesized CNPs was 0.9961. This value indicates that 99.61% of the variance in the biosynthesized CNPs was attributed to the independent factors; however, the model was only capable of describing 0.39% of the total variance. Table 2 displays the adjusted determination coefficient (Adj R^2^ = 0.9925) for the regression model of CNPs biofabrication; a higher value indicates more significance. High compatibility between observed and predicted values of the response was evidenced by the predicted R^2^ value = 0.9581, which was in reasonable agreement with the adj. R^2^ value^79^.

The model's mean, standard deviation and adequate precision are 4.29, 0.22 and 59.53, respectively (Table 2). Adequate precision indicates noise level; the level > 4 (59.53) is better and reveals high accuracy, suggesting appropriate design space for optimizing CNPs biofabrication at the various levels of the evaluated parameters^80^. Statistically analyzed data of CNPs biofabrication reveals that the coefficient of variation percent (C.V.) = 5.04% which is relatively low and reflects the high accuracy, reliability and precision of experimental trials^81^. Data also reveals a lower standard deviation (0.22).

In addition, the calculated coefficient showed that the independent factors have either positive or negative impacts on the biofabrication of CNPs. If the estimated effects are large, regardless of whether they are positive or negative, it can be concluded that the independent factors have a significant influence on the response. A positive sign for the coefficient of a tested variable indicates that increasing the variable value will increase production. In contrast, a negative sign indicates that production is greater when the variable has a low value^31,82^. There are two types of interactions that occur between two variables: antagonism (a coefficient with a negative value) and synergism (a coefficient with a positive value). Positive coefficients for X_1_ and X_3_ indicate that increasing the levels of these factors increases the biofabrication of CNPs. Furthermore, the negative oefficient value of X_2_ revealed that increasing the level of this factor reduces the biofabrication of CNPs.

Probability values (*P*-values) and *F*-values (Table 2) were used to determine the significance of each coefficient, which is necessary to assess the importance of the variables and interpret their interactions. The significance of the variable increased as the *P*-values decreased. In addition, process variables with *P*-values less than or equal to 0.05 were considered to have a significant impact on the response^83^. The model is statistically significant, with an *F*-value of 281.57 and a *P*-value of less than 0.0001. For chitosan nanoparticles biofabrication with *Lavendula angustifolia* leaves extract, the *P*-values of the coefficients indicate that the linear effects of initial pH level (X_1_), chitosan concentration (X_2_), and concentration of leaves extract (X_3_), interaction effects of X_1_ X_2_; X_2_ X_3_ and quadratic effect of X_2_ (chitosan conc.) are significant. Because of this, they function as limiting variables, and any change in their levels will cause a change in CNPs biofabrication.

Table 3 displays the fit summary results that used to establish which of the three polynomial models (linear, 2FI, and quadratic) was the best match for describing the biofabrication of CNPs by *Lavendula angustifolia* leaves extract. Where there is a non-significant lack of fit (*P*-value = 0.0923; *F*-value = 3.62), the quadratic model is the adequate model for CNPs biofabrication since it has higher values of the adj. R^2^ (0.9925) and predicted R^2^ values (0.9581). The given data demonstrates that the model's lack-of-fit error did not reach the significance level, as indicated by a higher *P*-value(*P*-value = 0.0923). The model also had an acceptable standard deviation, coefficient of variation, and degree of accuracy^84^.

**Table 3:** Fit summary of FCCCD for chitosan nanoparticles biofabrication using *Lavendula angustifolia* leaves extract.

Fit summary					
Source	Sequential *P* value	Lack of fit *P* value	Adjusted R^2^	Predicted R^2^	
Linear	< 0.0001	0.00	0.9623	0.9372	
2FI	0.0356	0.01	0.9755	0.9107	
Quadratic	0.0017	0.09	0.9925	0.9581	

Sequential model sum of squares					
Source	Sum of squares	*df*	Mean square	*F-*value	*P *value *P*rob > *F*
Linear versus Mean	115.06	3	38.35	162.78	< 0.0001*
2FI versus Linear	1.78	3	0.59	3.86	0.0356*
Quadratic versus 2FI	1.53	3	0.509	10.9	0.0017*

Lack of fit tests					
Source	Sum of squares	*df*	Mean square	*F-*value	*P-*value *P*rob > *F*
Linear	3.67	11	0.3335	16.48	0.0031*
2FI	1.89	8	0.24	11.69	0.0075*
Quadratic	0.37	5	0.07	3.62	0.0923

Model summary statistics					
Source	Standard deviation	R-Squared	Adjusted R-squared	Predicted R-squared	PRESS
Linear	0.49	0.9683	0.9623	0.9372	7.46
2FI	0.39	0.9832	0.9755	0.9107	10.61
Quadratic	0.22	0.9961	0.9925	0.9581	4.97

“*Significant values, *df* : degree of freedom, PRESS: sum of squares of prediction error, two factors interaction: 2FI”.

The following equation depicts the mathematical relationships between the independent variables and the results:

3 $${\text{Chitosan}}\;{\text{nanoparticles}}\;{\text{biofabrication}}\;{\text{value }} = \, + \, 4.03 \, + \, 0.45 \, X_{1} {-} \, 0.85 \, X_{2} + \, 3.25 \, X_{3} {-} \, 0.25 \, X_{1} X_{2} + \, 0.15X_{1} X_{3} - \, 0.37 \, X_{2} X_{3} - \, 0.09 \, X_{1}^{2} \, + \, 0.58 \, X_{2}^{2} \, + \, 0.03 \, X_{3}^{2}$$

where Y is the predicted value of CNPs biofabrication, initial pH level (X_1_), chitosan concentration (X_2_), concentration of leaves extract (X_3_).

### Effects of process variables on CNPs biofabrication using *Lavendula angustifolia* leaves extract (three-dimensional surface plots)

**Figure 6:**
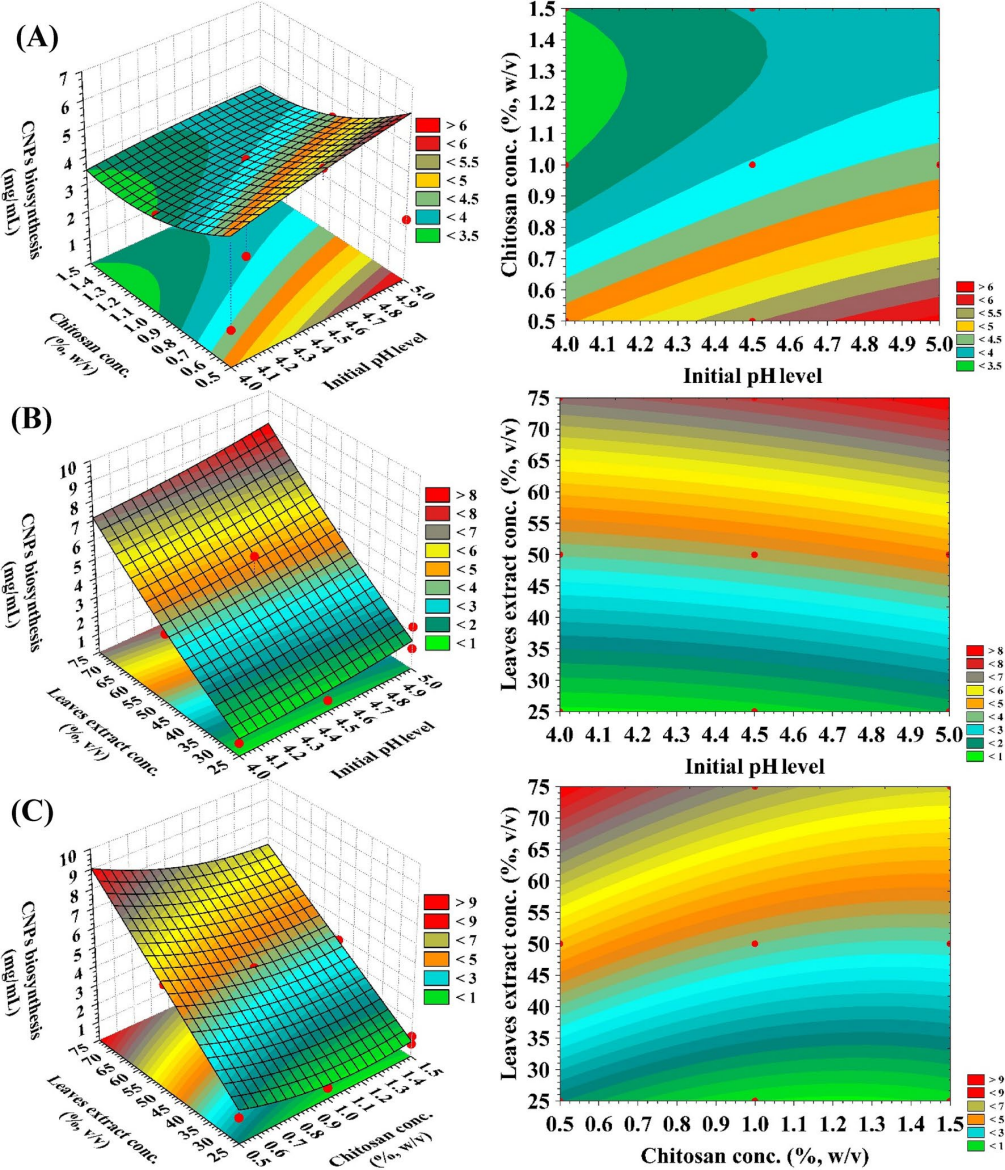
(**A**–**C**) 3D plots showing the mutual effects of initial pH level (X_1_), chitosan conc. (X_2_) and leaves extract conc. (X_3_) on CNPs bio-synthesized using *Lavendula angustifolia* leaves extract.

Three-dimensional (3D) surface plots were generated to examine the interaction effects of the three factors on the biofabrication of CNPs using *Lavendula angustifolia* leaves extract (Fig. 6).

### Effect of initial pH level on CNPs biofabrication

Three-dimensional response surface plots for the effect of initial pH on CNPs production using *Lavendula angustifolia* leaves extract are shown in Fig. 6A, B, where pH interacts with chitosan concentration (X_2_) and leaves extract concentration (X_3_); respectively.

According to the plots, an increase in the initial pH level led to a rise in the rate of CNPs production. The CNPs biofabrication was at its highest level (9.93 mg/mL) when the initial pH was raised to its highest level. According to the findings of Sathiyabama and Parthasarathy^37^, the optimal initial pH for maximum CNPs biofabrication was 4.8.

### Effect of chitosan concentration on CNPs biofabrication

The three-dimensional response surface plots for the effect of chitosan concentration (X_2_) on CNPs biofabrication as a function of initial pH level (X_1_) and the concentration of leaves extract (X_3_) are shown in Fig. 6A, C; respectively. According to the plots, the yield of CNPs biofabrication increased as the concentration of chitosan decreased to a lower level. The highest level of CNPs biofabrication (9.93 mg/mL) was achieved at a level of chitosan concentration that was significantly lower (about 0.5%) in the reaction mixture. Our finding is in accordance with those of Sathiyabama and Parthasarathy^37^ who reported that chitosan concentration of 0.5 percent was used for the synthesis of CNPs.

Vaezifar et al.^85^ found that a chitosan concentration at 1.5% was the best initial chitosan concentration to generate CNPs compared to greater concentrations. In contrast, Mahmoud et al.^86^ produced CNPs at a concentration of 2%. On the other hand, Kamat et al.^87^ stated that the highest production of nanoparticles could be obtained by using a concentration of chitosan of 0.8 mg/mL. The concentration of chitosan has a significant impact on both the size and production of the nanoparticles^88^.

### Effect of leaves extract concentration on CNPs biofabrication

The three-dimensional surface graphs depicting the interaction of the leaves extract concentration on CNPs biofabrication as a function of initial pH level (X_1_), chitosan concentration (X_2_); respectively (Fig. 6B and C). As shown the plot, there was a correlation between the rising the leaves extract concentration and the CNPs biofabrication. The yield of CNPs biofabrication was increased as the leaves extract concentration (percent) increased and the highest yield was achieved at around 75%.

### The model adequacy

**Figure 7:**
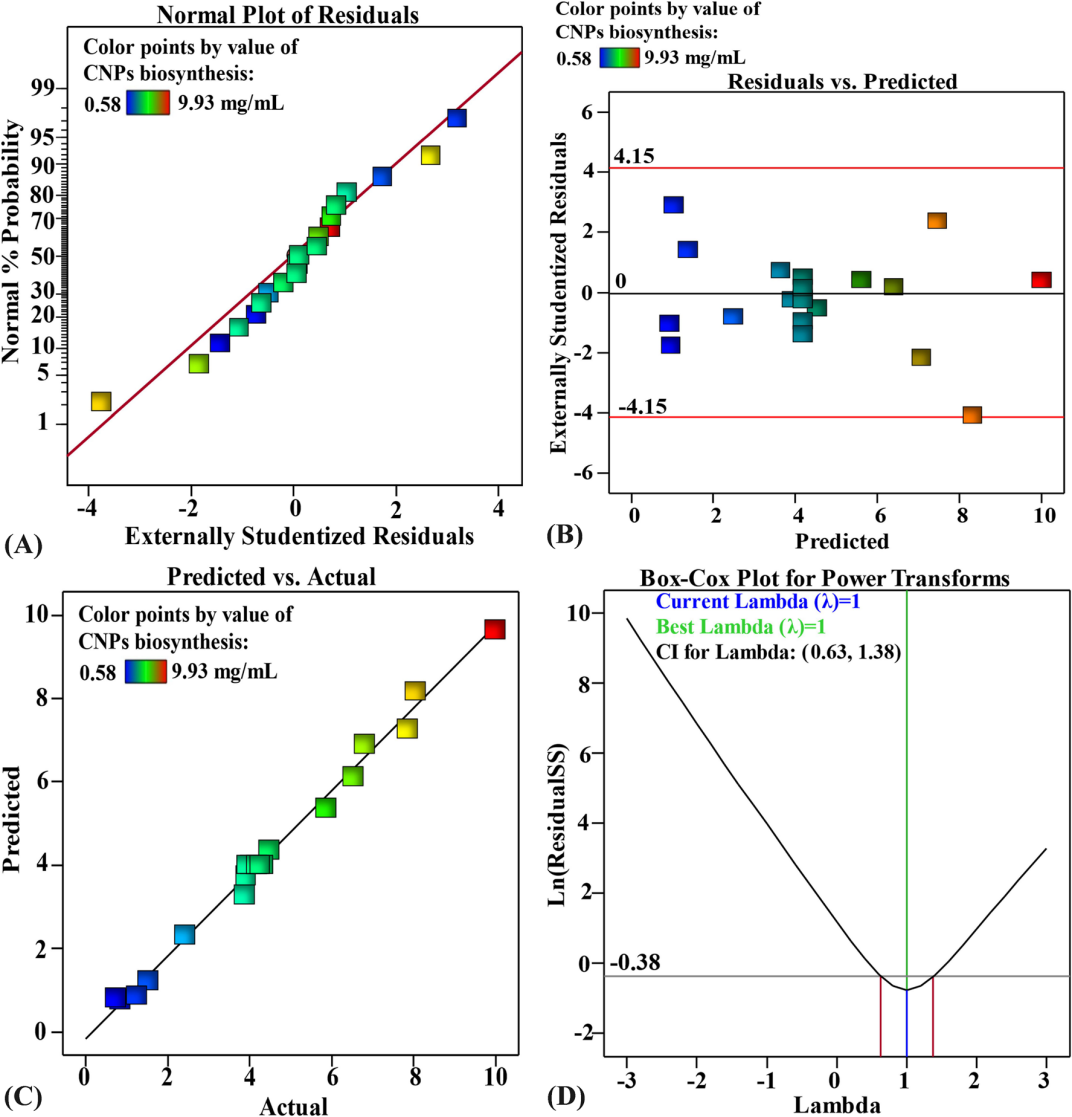
(**A**) Normal probability plot of internally studentized residuals, (**B**) plot of internally studentized residuals versus predicted values, (**C**) plot of predicted versus actual and (**D**) Box-Cox plot of model transformation of initial pH level (X_1_), chitosan conc. (X_2_) and leaves extract conc. (X_3_) on CNPs bio-synthesized using *Lavendula angustifolia* leaves extract.

The normal probability plot is a chart that indicates that the residuals should have an equal distribution in order to validate the fitness of the model^89^. Residuals are differences between theoretical predictions and experimental findings; a low value for these differences indicates that the model is accurate^90^. Figure 7A indicates that all of the points are located along the diagonal line, which suggests that the actual findings and the predicted data from the regression model are consistent with one another, which demonstrates that the model is accurate^34^. Figure 7B demonstrates that all of the residuals distributed uniformly around the zero line, indicating that the experimental data have a consistent variance^91,92^. The existing pattern of distribution is suitable enough to validate the FCCCD model. For CNPs biofabrication using *Lavendula angustifolia* leaves extract, the actual versus predicted values are shown in Fig. 7C, and all the points are positioned quite near to the prediction line, indicating acceptable fitting of the model to the experimental data^93^. Figure 7D depicts a Box-Cox plot of model transformation, which is useful for analysing non-normally distributed data. It demonstrates that the best Lambda (λ) value of 1 is between the two vertical red lines (representing the minimum and maximum values of the 95% confidence values, which are 0.63 and 1.38, respectively). This means that the model is a good fit for the experimental results acquired without any need for additional data manipulation^93^.

### ANN modelling prediction for CNPs biofabrication

**Figure 8:**
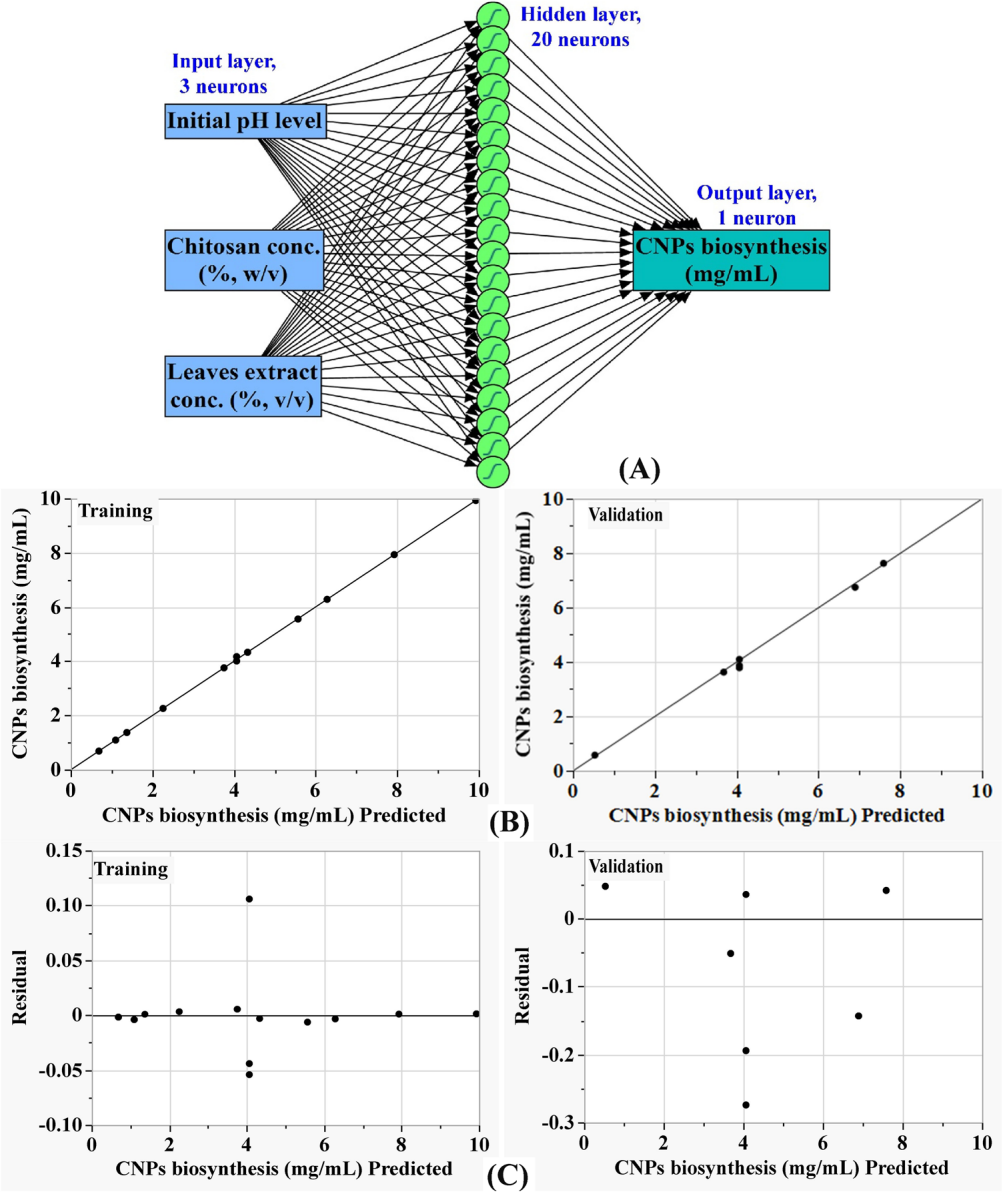
The final artificial neural network of the biosynthesized AuNPs (**A**), the ANN predicted versus actual (**B**), and the residuals versus ANN predicted (**C**) values of of CNPs bio-synthesized using *Lavendula angustifolia* leaves extract.

CNPs biofabrication by extract of *Lavendula angustifolia* leaves was analyzed, validated, and predicted using the artificial intelligence-based approach (Table 1). ANN is an advanced artificial intelligence technology instructs computers computers to build accurate and efficient models and analyses and interpret data like the human brain. The artificial neural network, also known as an ANN, is a technique for machine learning that is based on a network of interconnected units or nodes that are referred to as artificial neurons. These artificial neurons are meant to loosely model the neurons that are found in a biological brain. ANN architecture was constructed employing input neuron network topology in order to optimize the production of chitosan nanoparticles using extract from *Lavendula angustifolia* leaves. The artificial neural network used in this investigation has one input layer that is composed of the three independent variables (initial pH level, chitosan concentration, the leaves extract concentration). Input nodes process the data, analyze or categorize it, and pass it to the subsequent layer. Hidden layer with 20-neurons gets their data from the input layer. One output layer provides the final outcome of the artificial neural network's data processing (CNPs biofabrication, mg/mL) (Fig. 8A). The optimal ANN parameters were adjusted to number of tours (5000), model NTanH (20), a learning rate of 0.1 and a validation method (holdback, 0.2), confidence intervals (1) and transform covariates (1). Machine learning was carried out until training and validation error metrics, including mean absolute deviation (MAD), root mean square error (RMSE), and sum of squared errors (SSE) were all at their minimum possible values, as well as the greatest value of R^2^, for both training and validation processes (Table 4).

**Table 4:** ANN analysis and modeling comparison of predictive capability between FCCCD and ANN for CNPs biofabrication using *Lavendula angustifolia* leaves extract.

Measure	ANN		Overall model performance		
	Training	Validation	Statistics	FCCCD	ANN
R^2^	0.9998	0.9956	R^2^	0.9960	0.9987
RASE	0.0353	0.1420	RASE	0.1538	0.0887
MAD	0.0180	0.1125	AAE	0.126	0.0511
SSE	0.0162	0.1412	Freq	20	20
Sum Freq	13	7			

MAD; mean absolute deviation, SSE; the sum of squares error, RASE; root average squared error, AAE; average absolute error for each model.

### Evaluation of ANN model

CNPs biofabrication predictions by ANN for the experimental results are listed in Table 1. A comparison was made between the actual values of CNPs biofabrication and the values that were predicted by ANN (Fig. 8 B). Both throughout the training phase and the validation phase, the points are getting closer to the line that represents the ideal prediction, which is an indication that the model is accurate. Figure 8 C shows that the residuals are normally distributed, both above and below the regression line, providing more evidence for the validity of the ANN model.

### Comparison of prediction potential of ANN versus FCCCD

The performance of the ANN versus FCCCD was evaluated with the help of the Model comparison dialog found in JMP Pro14. Table 1 demonstrates that in comparison to the FCCCD model, the ANN model's predictions for CNPs biofabrication exhibit better agreement with the experimental results and have lower residuals. The predictive efficacy of the FCCCD and the ANN was compared using the R^2^ as well as root average squared error (RASE), and average absolute error (AAE) (Table 4). Table 4 demonstrates that the ANN is the superior design due to its greater ability to predict the optimum levels of the selected variables. This is supported by the higher R^2^ value (0.9987) of the ANN model and the lower RASE and AAE values of 0.0887 and 0.0511; respectively.

**Figure 9:**
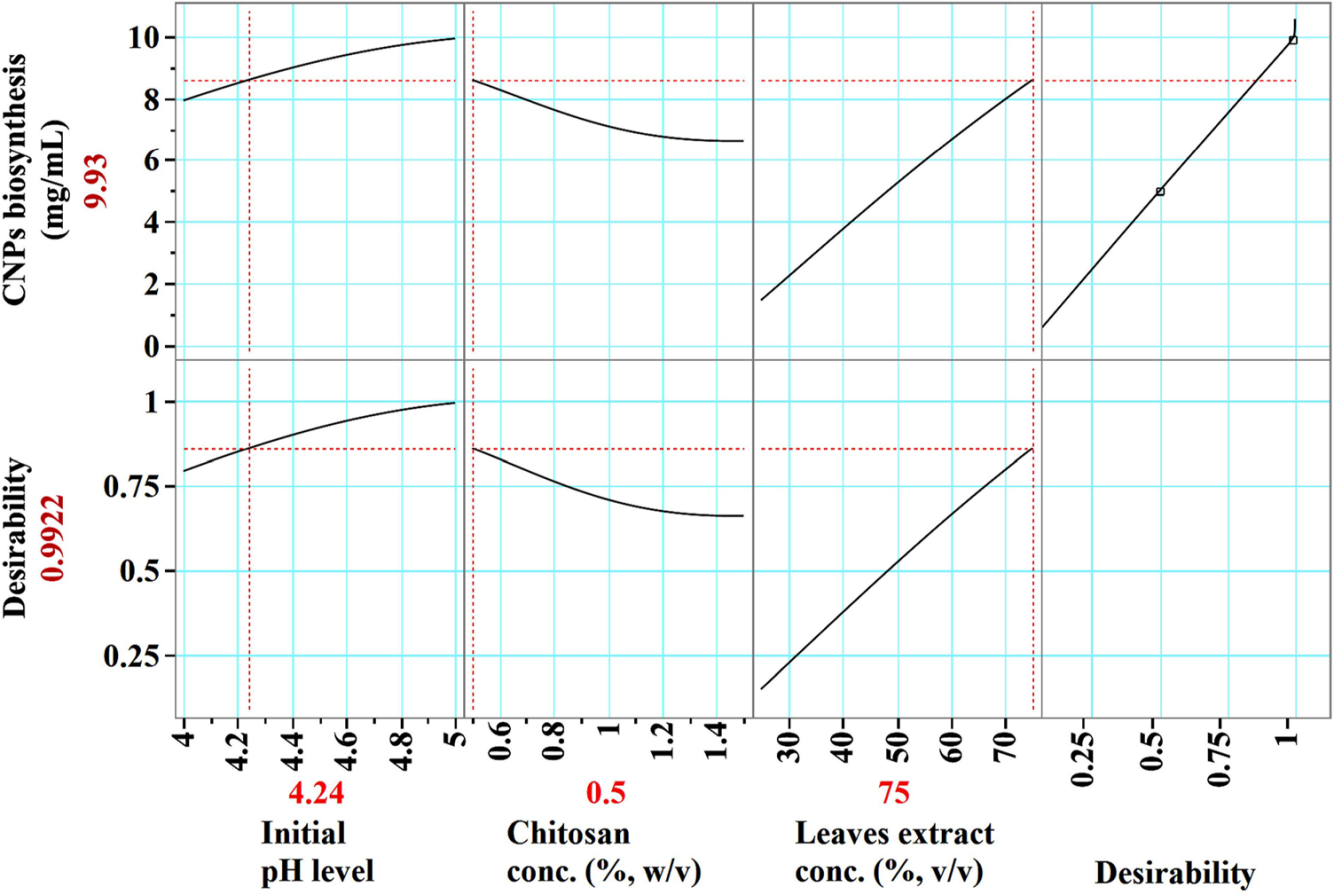
The optimization plot displays the desirability function and the optimum predicted values of of CNPs bio-synthesized using *Lavendula angustifolia* leaves extract.

The desirability function, which can be seen in Fig. 9, was carried out in order to establish the most accurate predictions of the conditions that would result in the highest possible value for CNPs production^94^. The highest predicted value of CNPs by ANN was 9.93 mg/mL at an initial pH of 4.24, a chitosan concentration of 0.5%, and leaves extract concentration of 75%. An experimental validation of the optimization strategy is carried out. The highest experimental value of CNPs biofabrication using *Lavendula angustifolia* leaves extract was proven to be 10.11 mg/mL under the previous conditions, and the finding was compared to the value predicted by ANN, which was 9.93 mg/mL. Results from the validation showed a high degree of model accuracy, demonstrating the model's reliability at the selected factor levels.

### Antibiofilm activity of CNPs

In the natural milieu, microorganisms display prominently diverse and complicated social coordination and cooperations. Majority of them have the ability to switch their lifestyle from free-floating mode to sessile community covered entirely with extracellular polymeric substance (EPS) scaffold, which is irreversibly associated with biotic and abiotic surfaces. In this life mode, different levels of genotypic, morphotypes and phenotypic heterogeneity are expressed with inter- and intra-species interaction and intense quorum signals. Such architecture furnishes the microbial cells with a highly protective system against harsh circumstances and various biocidal agents. By such way, the microbial biofilms menace public health and the whole ecosystem at environmental, medical, pharmaceutical and industrial sectors^7^. Subsequently, diverse mechanical and chemical methods were applied to handicap biofilm dissemination^95^. Recently, metal nanotechnology involved strongly in commercial production of synthetic antiadhesive and antibiofilm agents. Nonetheless, the unaccounted cytotoxicity and ecotoxicity impacts of metal nanoparticles and also the possibility of prevalence of multi-drug resistance phenomena could restrict their wide application.

**Figure 10:**
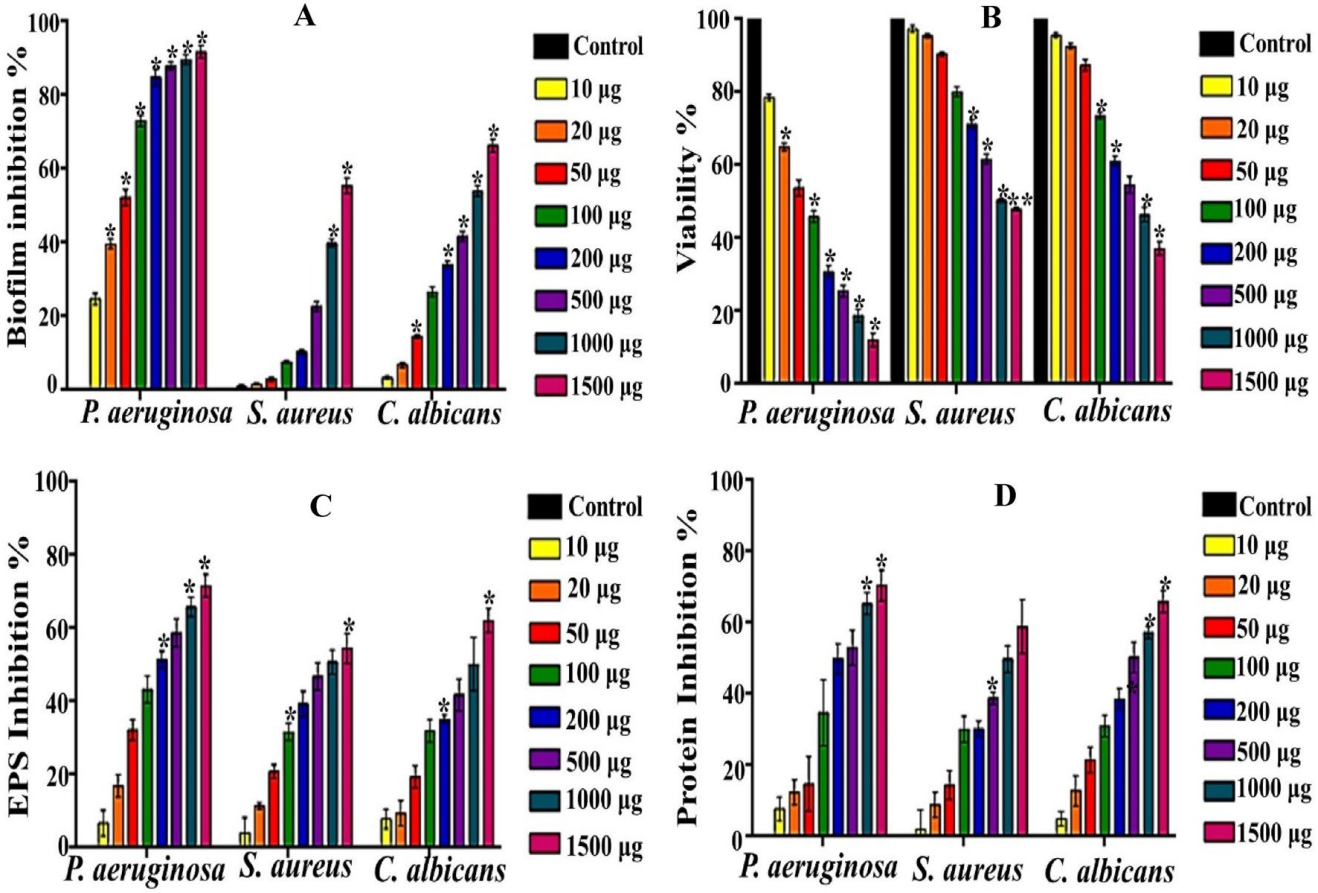
The impact of CNPs on biofilm growth by *P. aeruginosa, S. aureus* and *C. albicans*. A-antibiofilm activity, B-Metabolic activity, C- EPS suppression and D-protein suppression. All values were represented as mean ± SEM. Treatments at different concentrations were compared with control with significance at *P < 0.05.

However, the prompt advancement in utilization of natural polymers conjugated with bionanotechnology has gained a momentum in safe, biocompatible, economic and influential treatment of pathogenic biofilms, which agreed with goals of recent international events like COP27. Accordingly, the antibiofilm activity of CNPs at different concentrations was investigated, in response to *P. aeruginosa*, *S. aureus* and *C. albicans* as representative strains of gram-negative, gram-positive bacteria and eukaryotic biofilm-forming pathogens, respectively. Their pathogenicity was listed tremendously in food intoxication, water-borne diseases and nosocomial infections^96,97^. Herein, crystal violet was employed to detect the biofilm inhibition directly on the bottom and inner walls of the microtiter plate. Notably, for all concentrations of CNPs, the biofilm formation, in all examined pathogens, was diminished compared to the control in a concentration-dependent inhibition manner. Additionally, the capability of CNPs to cease the biofilm formation was varied significantly among the examined strains, reflecting differences of biofilm structural characteristics based on microbial type, growth conditions and nutrients abundancy^98^. Apparently, *P. aeruginosa* was the most pathogen influenced adversely by all tested concentrations of CNPs. As noticed in Fig. 10A, low concentration (10–50 μg/mL) suppressed biofilm synthesis of *P. aeruginosa* by 24.7 ± 1.52% – 52.26 ± 2.1%. Whereas, at exact concentrations, CNPs inhibited *S. aureus* by 1.14 ± 0.196%—3.03 ± 0.631%; reflecting higher tolerance of *S. aureus*. Regarding the biofilm of *C. albicans*, moderate reduction in biofilm proliferation was observed at 10–50 μg/mL of CNPs by the range of 3.47 ± 0.48 to 14.4 ± 0.48%. Remarkably, as denoted by ANOVA, significant (*P* ≤ 0.05) and more evident inhibition was exerted by 1500 μg/mL of CNPs against *P. aeruginosa*, *S. aureus* and *C. albicans* by 91.83 ± 1.71%, 55.47 ± 2.12% and 66.4 ± 1.76%, respectively. Therefore, the potentiality of CNPs to antagonize the biofilm-forming pathogens could also be portrayed as microbe-dependent. In comparison, Yien et al.^99^ found that MIC of CNPs against *C. albicans* recorded 0.6–0.85 mg/L based on the molecular weight of bulk chitosan; reflecting advantageous feature of our CNPs in candidiasis therapy, in particular among immunocompromised patients. On the other hand, Aguayo et al.^100^ and Pan et al.^101^ recorded that 280 μg/mL and 75 μg/mL of CNPs suppressed *P. aeruginosa* and *S. aureus* biofilm formation by 88.9% and 85.8%, respectively.

Regarding the antibiofilm activity of *Lavendula angustifolia* extract, the results showed its inhibitory effect on the biofilm development of *P. aeruginosa*, *S. aureus* and *C. albicans* by 10.48 ± 2.22, 24.77 ± 2.72 and 14.57 ± 3.1%, respectively. Generally, such antimicrobial potency was recorded by different research groups; ascribing that to the availability of alkaloids, flavonoids and essential oils in the plant extract^102^. It is important to mention that the antibiofilm potency of CNPs attributed utterly to the CNPs impact, as they washed and purified several times from any extract residues during processing step.

### Impact of CNPs on biofilm metabolism and biochemical constituents

The respiratory activity of cells within biofilm matrix after 24 h incubation was evaluated by using MTT reduction assay Fig. 10B. As observed previously, the different doses of CNPs restricted progressively the growth and metabolic performance of the examined strains. Wherein, the most potent and significant metabolic activity inhibition was noticed against *P. aeruginosa* at the highest concentration (i.e., 1500 μg/mL) by 87.84 ± 1.83%, comparing to 21.39 ± 0.83% at 10 μg/mL. On the other hand, lower capability of CNPs at 10 μg/mL in blocking cell viability was observed in the cells of *S. aureus* and *C. albicans* biofilms by 2.69 ± 0.95% and 4.4 ± 0.72%, respectively. Notably, the inhibitory power of CNPs increased in lessening the oxidative activity upon elevating their concentration to 1500 μg/mL, which recording 52 ± 1.485% and 62.76 ± 1.89% for *S. aureus* and *C. albicans* biofilms, respectively.

As well know, the main defensive barrier of the biofilm is the three-dimensional sticky complex matrix (i.e., EPS), which contains more than 90% of polysaccharides (e.g., cellulose nanofibers, mannose, rhamnose, arabinose, sucrose-derived glucans and fructans) and proteins (e.g., lectins, Baplike proteins and curli fimbriae)^103^. Interestingly, the ratios of biofilm chemical constituents differ among various microbial species and influenced by environmental factors^104^. Accordingly, the impact of different concentrations of CNPs on carbohydrate and protein contents of biofilm was studied. As demonstrated in Fig. 10C, a significant reduction (*P* ≤ 0.05) in EPS content of *P. aeruginosa*, *S. aureus* and *C. albicans* biofilms was recorded by 71.74 ± 3.06, 54.53 ± 4.185 and 62.19 ± 3.24%, respectively. As its synthesis was inhibited from 45.84 ± 0.28, 30.66 ± 1.02 and 26.50 ± 1.38 mg/mL in untreated control samples and reached to 12.96 ± 1.41, 13.94 ± 1.28 and 10.02 ± 0.86 mg/mL in samples treated with 1500 ug/mL of CNPs, respectively. Likewise, Fig. 10D illustrated significant (*P* ≤ 0.05) and remarkable changes in protein content of *P. aeruginosa*, *S. aureus* and *C. albicans* biofilms caused by 1500 ug/mL of CNPs. Where their protein content declined from 16.28 ± 0.42, 16.09 ± 1.0 and 9.51 ± 1.15 mg/mL to 4.80 ± 0.7, 6.61 ± 1.21 and 3.24 ± 0.3 mg/mL with inhibition percentages reached 70.52 ± 4.30, 54.95 ± 7.55 and 65.98 ± 3.11%. intriguingly, the agreement in results of antibiofilm, viability and biochemical constituents of the examined biofilms indicated the effectiveness of CNPs in suppressing biofilm formation, affecting negatively on their components and impairing the metabolic activity of adherent cells.

### Ultrastructure study of biofilms upon CNPs treatment:

**Figure 11:**
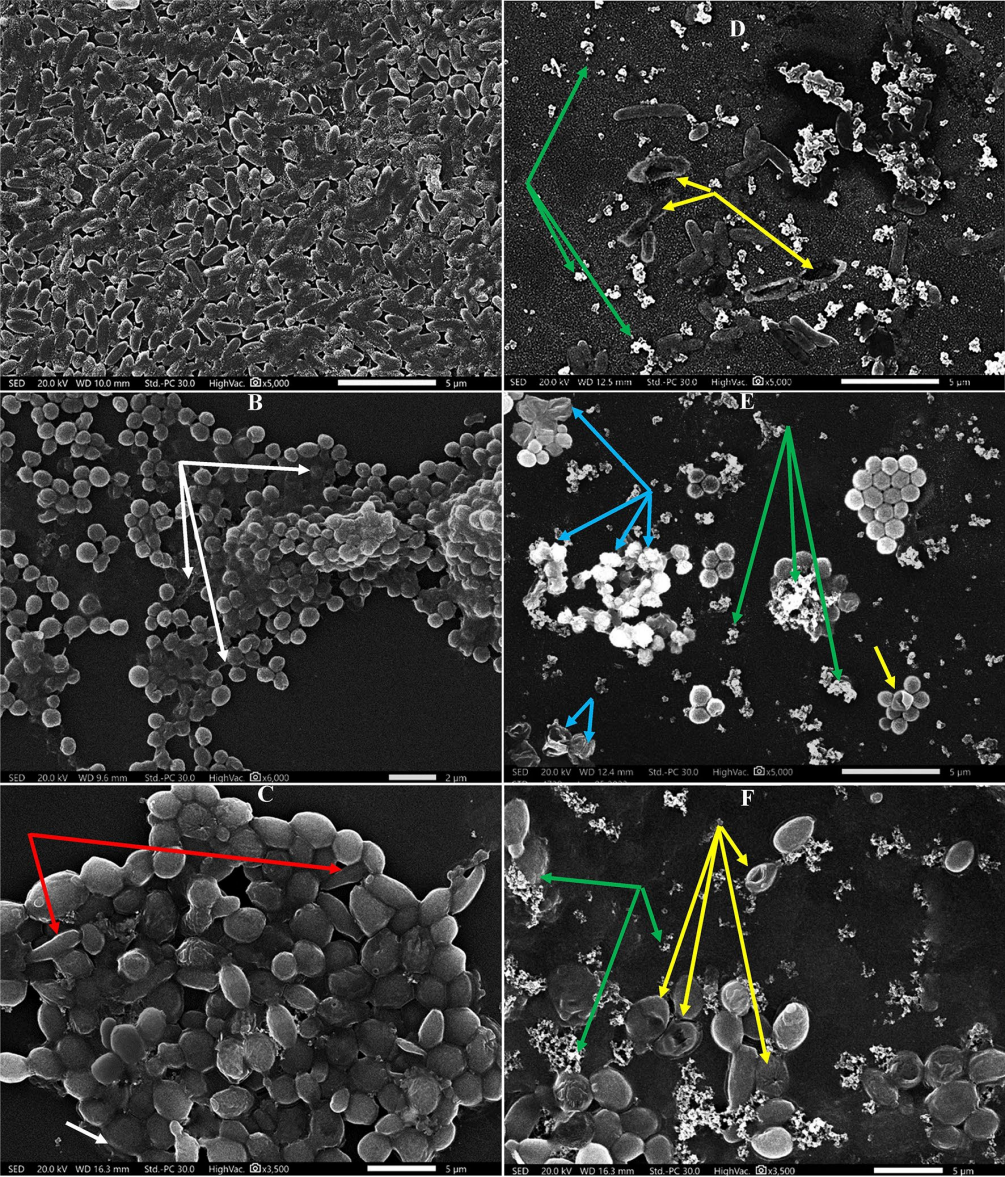
SEM micrographs depicting the effect of CNPs on *P. aeruginosa* (**A**&**D**)*, S. aureus* (**B**&**E**) and *C. albicans* (**C**&**F**) biofilms. Left panel represents untreated control samples, while right panel represents treated samples. White arrows: EPS, red arrows: early-stage of hyphae formation, green arrows: CNPs aggregates, yellow arrows: furrows, dimples and indentations and blue arrows: ghost cells.

Herein, the employment of complementary microscopic tool like SEM empowered a deeper vision to visualize the morphological changes consequential to CNPs treatment on architecture properties of *P. aeruginosa*, *S. aureus* and *C. albicans* biofilms. As depicted in Fig. 11, the biofilm morphology and structure of each studied microbe appeared unique and different in cell texture, cell shape/size and their distribution inside the EPS-matrix. Nonetheless, they shared common features in the control untreated samples. Wherein, they showed healthy morphology with smooth cell surface, regular cell boundaries and normal size estimated by 1.2 ± 0.2 μm length and 0.2 ± 0.05 μm width for *P. aeruginosa*; around 0.35 ± 0.07 μm and 2.5 ± 1.1 μm in diameter for *S. aureus* and *C. albicans*, respectively. Besides, rods of *P. aeruginosa* appeared compactly arranged in monolayer structure of slimy EPS (Fig. 11A). Whereas, multilayer clumps of *S. aureus* spheres were wrapped with dense viscous EPS matrix (White arrows, Fig. 11B). Meanwhile, round to oval shaped blastospores with budding and early-stage of hyphae (referred by red arrows) of *C. albicans* were tightly packed in homogenous mucilaginous architecture as evident in Fig. 11C. Interestingly, the association of hyphae and pseudo-hyphae stages with yeast cells strengthen biofilm structure and symbolizes as potent virulence factor during infection process^105,106^.

Upon CNPs treatment, obvious reduction in biofilm mass, alterations in cells size, diminishing biofilms density with deformed texture were noticed, particularly in *P. aeruginosa* biofilm. As demonstrated in Fig. 11D, the loosely packed cells became more elongated and scattered separately in EPS matrix, which appeared completely destructed by CNPs aggregates attached to the cells (pointed out by green arrows). In the same sense, Horst et al.^107^ observed the aggregation behavior of metal NPs on the biofilm’s surface. Let alone detecting wide furrows in the cells (referred by yellow arrows); implying dramatic deterioration of the cell’s membranes, leakage of intracellular cytoplasmic components and losing the ability for dividing properly and forming EPS lattice as reported by Krishnamurthi et al.^108^. Similarly, Zhou et al.^109^ stated the same damage traits in *P. aeruginosa* biofilm when treated with antibiofilm agents. On the other hand, a moderate damage was visualized in *S. aureus* biofilm. Despite most of the cells retained their ordinary shape and size, a lower number of cells were individually dispersed in decayed EPS envelope. Notably, clumps of disintegrated cells with unspecified shape, rough surface and lysed cell lines seemed to be aggregated in a phenomenon called ghost cells (pointed out by blue arrows). Besides, small indentations were also observed on cell surfaces as referred by yellow arrows (Fig. 11E). Likewise, Eltarahony et al.^110^, documented the same destructive features in *S. aureus* by the action of NPs. Concerning *C. albicans* biofilm, the exact magnification of both control and treated samples revealed low number of deformed cells with groves, dimples and ruffled surfaces in disrupted integrity of EPS (Fig. 11F). Similar features in *C. albicans* biofilm deformation induced by different fungicides were mentioned by other studies^111–113^.

Arguably, the current study unveiled the growth suppression capability and biofilm destabilization potency of CNPs against the protective EPS skeleton of studied biofilm, which could be ascribed to the polycationic nature of chitosan and its nanoscale formulation. Wherein, abundance of the amino groups (NH^3+^) of N-acetylglucosamine units of chitosan molecule facilitates the electrostatic binding with negatively charged moieties, which are disseminated on microbial cell membranes^114^. Hereby, CNPs influenced on of the physical–chemical properties of biofilm, namely, polymeric properties, hydrophobicity and hydrophilicity, which eventually destabilized the formation and adhesion of biofilm^115,116^. Besides, metal-chelating property of chitosan, which allow capturing of essential metals from surrounding ambient led to chitosan-metal-complex formation; preventing by such way the flow and integration of metal ions in their corresponding active sites at the functional groups of essential biomolecules; ultimately, lethal damage for microbial cells. Interestingly, CNPs of this study serve in dual function, namely, external outer membrane disruptor and internal penetrator, by the virtue of their nanoscale diameter. Such character furnished chitosan higher surface area and more penetration capability into biofilm structure through porins channels; subsequently extra cellular injuries^114,117^.

Strikingly, a potent and sever deformation was displayed by gram-negative biofilm than gram-positive as shown in SEM micrographs, which validated the results of antibiofilm, viability and biochemical constituents. That could be attributed to the higher hydrophobicity of gram-negative bacteria and also the affluency of negatively-charged functional groups of lipopolysaccharides and phospholipids which adsorb more cationic chitosan charges; ultimately more antagonistic potency. On the other hand, lower density of negatively charged functional groups of thick peptidoglycan layer with low hydrophobicity enable gram-positive bacteria to tolerate lethal effect of chitosan^114^. Contrary, Coma et al. ^118^ and Dutta et al.^119^ reported the higher susceptibility of *Listeria monocytogenes, Lactobacillus plantarum, B. cereus* and *Staphylococcus aureus* to chitosan than *E. coli, Salmonella typhymurium* and *Vibrio parahaemolyticus*. Meanwhile, a promising fungicidal activity was noticed by CNPs of the current study in defeating *C. albicans* biofilm. That could be attributed to the enhanced membrane fluidity of *Candida* species, on particular, owing to the availability of negatively charged unsaturated fatty scattered on fungal cell surface^120^. Otherwise, the fungistatic performance was assigned to CNPs rather than fungicidal as described by Rabea et al.^121^ and Goy et al.^114^. However, the contradiction in scholars finding regarding to biocidal activity of CNPs against different pathogens could be assign for different reasons, which are dedicated to the traits of CNPs (e.g., shape, size, surface charge, etc.), nature of treated microbe (e.g., microbial load, type, physiology, etc.) and treatment milieu (e.g., treatment period, pH, temperature, etc.)^122^.

Generally, the antibiofilm potentiality of CNPs could be displayed during all phases of biofilm formation. In planktonic phase, the CNPs sheath surrounding the cells inhibits biofilm formation via deterioration of cell membrane integrity, increasing wall permeability, triggering osmotic imbalances and elevating the infiltration rate of intracellular cytoplasmic constituents. Upon the subsequent phase of cell adhesion, CNPs coat prohibits EPS production, destabilizes irreversible EPS attachment to biotic or abiotic surfaces and reducing hydrophobicity. Besides, CNPs bind tightly to microbial biomolecules such as eDNA, RNA and amino acids; causing blocking of their functions. Moreover, the cell-to-cell signaling strategy (i.e., quorum sensing) was also interrupted by CNPs ^117^. Despite CNPs effectiveness, Dhillon et al.^96^, Atay et al*.*^123^ and Muthuchamy et al.^124^ empowered the biological activity of CNPs by combining with other natural biomolecules, antibiotics, metal nanoparticles and graphene.

## Data Availability

All data generated or analyzed during this study are included in this article.
